# A tailored series of engineered yeasts for the cell-dependent treatment of inflammatory bowel disease by rational butyrate supplementation

**DOI:** 10.1080/19490976.2024.2316575

**Published:** 2024-02-21

**Authors:** Jiahao Wu, He Huang, Lina Wang, Mengxue Gao, Shuxian Meng, Shaolan Zou, Yuanhang Feng, Zeling Feng, Zhixin Zhu, Xiaocang Cao, Bingzhi Li, Guangbo Kang

**Affiliations:** a Frontiers Science Center for Synthetic Biology and Key Laboratory of Systems Bioengineering (Ministry of Education), School of Chemical Engineering and Technology, Tianjin University, Tianjin, China; b Department of Gastroenterology and Hepatology, Tianjin Medical University General Hospital, Tianjin, China; c Frontiers Research Institute for Synthetic Biology, Tianjin University, Tianjin, China

**Keywords:** Inflammatory bowel disease, butyrate production, saccharomyces cerevisiae, synthetic biology, gut microbiota

## Abstract

Intestinal microbiota dysbiosis and metabolic disruption are considered essential characteristics in inflammatory bowel disorders (IBD). Reasonable butyrate supplementation can help patients regulate intestinal flora structure and promote mucosal repair. Here, to restore microbiota homeostasis and butyrate levels in the patient’s intestines, we modified the genome of *Saccharomyces cerevisiae* to produce butyrate. We precisely regulated the relevant metabolic pathways to enable the yeast to produce sufficient butyrate in the intestine with uneven oxygen distribution. A series of engineered strains with different butyrate synthesis abilities was constructed to meet the needs of different patients, and the strongest can reach 1.8 g/L title of butyrate. Next, this series of strains was used to co-cultivate with gut microbiota collected from patients with mild-to-moderate ulcerative colitis. After receiving treatment with engineered strains, the gut microbiota and the butyrate content have been regulated to varying degrees depending on the synthetic ability of the strain. The abundance of probiotics such as *Bifidobacterium* and *Lactobacillus* increased, while the abundance of harmful bacteria like *Candidatus Bacilloplasma* decreased. Meanwhile, the series of butyrate-producing yeast significantly improved trinitrobenzene sulfonic acid (TNBS)-induced colitis in mice by restoring butyrate content. Among the series of engineered yeasts, the strain with the second-highest butyrate synthesis ability showed the most significant regulatory and the best therapeutic effect on the gut microbiota from IBD patients and the colitis mouse model. This study confirmed the existence of a therapeutic window for IBD treatment by supplementing butyrate, and it is necessary to restore butyrate levels according to the actual situation of patients to restore intestinal flora.

## Introduction

Inflammatory bowel disease (IBD) is characterized by the inflammation of the gastrointestinal tract. The primary forms of IBD include Crohn’s disease and ulcerative colitis^1^. The current predicament in IBD treatment relates to the intricate nature of the disease and the absence of a universally effective treatment.^2,3^ Treatment of IBD often entails a combination of pharmaceutical interventions, lifestyle adjustments, and in some cases, surgical procedures. Nevertheless, determining the most appropriate treatment approach for each individual is challenging owing to the varying nature of the disease and the potential adverse effects of medications. Furthermore, achieving long-term remission and the effective management of flare-ups continue to pose significant obstacles in the treatment of IBD.

The conventional medications used for the treatment of IBD include anti-inflammatory drugs, immunosuppressants, and biological agents. Nonetheless, these medications can elicit various side effects, such as indigestion, nausea, osteoporosis, muscle degeneration, susceptibility to infections, abnormal liver function, immunosuppression, and increased risk of cardiovascular issues.^4–6^ Butyrate, recognized for its anti-inflammatory properties,^7^ is a potential medication for IBD treatment. Butyrate plays a vital role in reinstating the function of the intestinal barrier, regulating immune response, and promoting balanced gut microbiota.^8,9^ It is a short-chain fatty acid (SCFA) produced via fermentation by butyrate-producing bacteria in the intestine. However, patients with IBD have been reported to have reduced levels of butyrate^10^ and a decrease in the abundance of butyrate-producing bacteria.^11^

Currently, various methods exist for supplying butyrate to a patient’s colon, including oral administration, supplementation with natural butyrate-producing bacteria, microencapsulation for butyrate delivery, and targeted technology for specific site delivery of butyrate.^12,13^ Regrettably, oral butyrate has drawbacks such as unpleasant odor, taste, and limited bioavailability.^14^ In addition, most butyrate-producing bacteria cannot directly utilize dietary fiber and require cross-feeding with other bacteria,^15^ rendering strains derived solely from the intestinal microenvironment ineffective. Furthermore, fecal microbiota transplantation lacks sufficient evidence for both safety and efficacy.^16,17^ The use of microencapsulation and targeted technology for butyrate release into the intestinal tract presents challenges, including high production costs and complex manufacturing processes. Therefore, there is an urgent demand for a novel delivery technology that can effectively transport butyrate to the intestinal tract of patients with IBD.

Probiotics are living microorganisms that, when consumed in sufficient quantities, provide health benefits to the host.^18^ Substantiated evidence demonstrates the essential role of probiotics in reinstating and sustaining a balanced gut microbiota, fortifying gut barrier function, regulating immune responses, and enhancing nutrient absorption.^19,20^ Advances in synthetic biological tools and genome-editing technologies have facilitated the engineering and modification of probiotics to improve stress tolerance, target specific pathogens, and facilitate the targeted delivery of drugs, such as antineoplastic drugs and insulin, to specific organs.^21–23^ *Saccharomyces cerevisiae*, which is used as a probiotic, serves as a platform for natural product synthesis and has extensive applications in the fields of food and medicine.^24–26^ Scott^27^ described the development of an engineered *S. cerevisiae* strain that secretes an enzyme capable of degrading adenosine triphosphate (ATP) in the gut, effectively inhibiting intestinal inflammation and reducing intestinal fibrosis in various mouse models of IBD. Sun^28^ reported that lactic acid-producing *S. cerevisiae* mediated the suppression of macrophage pyroptosis and modulation of intestinal flora to mitigate colitis. Hence, synthetic biology can be employed to synthesize butyrate within probiotics such as *S. cerevisiae* for the treatment of IBD. Nevertheless, the characteristics of the intestine, particularly the distribution of oxygen in the colon,^29^ necessitate the consideration of engineered probiotic tolerance to the varying oxygen concentrations in the environment. It is crucial to acknowledge that high levels of butyrate can lead to adverse effects such as the induction of oxidative stress,^30^ highlighting the importance of controlling the quantity of butyrate released by engineered yeast.

**Figure 1.**
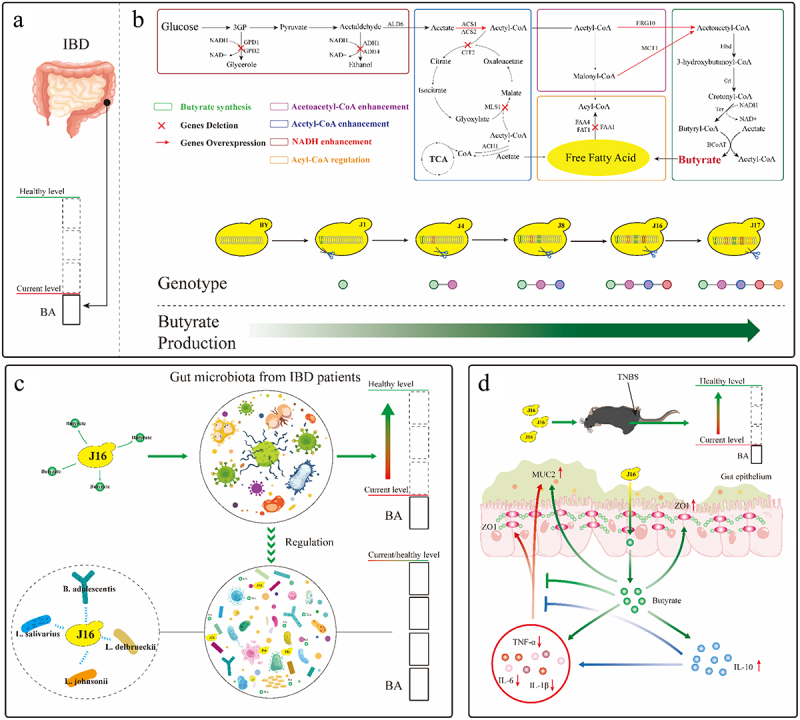
Constructing butyrate producing yeasts for the treatment of IBD.

A large number of clinical trials have shown that the levels of short-chain fatty acids, especially butyrate, in the intestinal cavity of IBD patients are significantly reduced compared to healthy individuals.^31,32^ We detected and compared the content of butyrate in fecal samples of IBD patients and healthy individuals, confirming that the occurrence of intestinal inflammation does indeed affect the generation of butyrate (Figures 5(b) and 1(a)). In this study, we used synthetic biological techniques to engineer *S. cerevisiae* to produce butyrate (Figure 1(b)). Careful consideration was given to selecting optimal enzymes capable of converting acetoacetyl-CoA into butyrate, considering that wild yeast cannot naturally synthesize butyrate. By evaluating the contribution of different exogenous butyrate-synthesis genes to yeast butyrate production, we constructed a recombinant *S. cerevisiae* strain capable of biosynthesizing butyrate through the heterologous expression of four key genes. Additional metabolic modules were incorporated to increase butyrate production in the engineered yeast strain. An acetoacetyl-CoA enhancement module was introduced to increase the availability of acetoacetyl-CoA, which served as a substrate for butyrate production. An acetyl-CoA enhancement module was integrated to amplify the supply of acetyl-CoA, a precursor for butyrate biosynthesis, particularly under anaerobic conditions. A nicotinamide adenine dinucleotide (NADH) enhancement module was implemented to increase NADH availability, thereby enhancing butyrate production in the engineered yeast strain under anaerobic conditions. Finally, an acyl-CoA regulation module was introduced to regulate fatty acid synthesis and minimize the metabolic consumption of butyrate. These integrated metabolic modules collectively contributed to the optimization of butyrate production in the engineered yeast strain. Following an assessment of butyrate production by various engineered yeast strains under different levels of oxygen, we tested the therapeutic effects of these strains on the gut microbiota derived from patients with IBD, as well as in a trinitrobenzene sulfonic acid (TNBS)-induced enteritis model (Figure 1(c,d)). By comparing the therapeutic outcomes of different engineered yeasts, we identified a strain called BYJ16 that exhibited the most favorable butyrate synthesis capability and demonstrated the best therapeutic effects on gut microbiota homeostasis in patients with IBD and mice with colitis. Previous studies have shown that butyrate in the human gut is crucial for maintaining the structure of gut microbiota and maintaining the homeostasis of gut microbiota.^33,34^ Butyrate is closely related to intestinal probiotics, and stable levels of butyrate can enrich intestinal probiotics such as *Lactobacillus* and *Bifidobacterium*.^35–37^ In in vitro experiments, the engineered yeast BYJ16 improved the structure of the gut microbiota and greatly increased the abundance of probiotics such as *Lactobacillus* and *Bifidobacterium* (Figure 1(c)). Numerous studies have shown that butyrate can inhibit the generation of inflammatory factors by mediating G protein-coupled receptors or inhibiting HDAC activity.^38–44^ In in vivo experiments, BYJ16 exhibited the most significant improvement in the symptoms of colitis in mice, along with reducing the generation of pro-inflammatory factors and enhanced gut barrier function by restoring butyrate levels in the intestinal tract of mice (Figure 1(d)).

(a) Research shows that the content of butyrate in the intestinal cavity of IBD patients is lower than that of normal individuals.^31,32^ BA, butyrate. (b) Based on *S. cerevisiae*, reconstruct butyrate synthesis pathway and regulate metabolism modules to construct a series of engineering yeasts with different butyrate production capacity. ERG10, acetyl-CoA C-acetyltransferase; MCT1, malonyl-CoA: ACP transferase; MLS1, malate synthase; CIT2, citrate synthase; ACS1, ACS2, acetyl-CoA synthetase; ALD6, cytosolic aldehyde dehydrogenase; ACH1，acetyl-CoA hydrolase; ADH1, ADH4, alcohol dehydrogenase; GPD1, GPD2, glycerol-3-phosphate dehydrogenase; FAA1, FAA4, Long chain fatty acyl-CoA synthetase; FAT1, very long chain fatty acyl-CoA synthetase and fatty acid transporter. (c) The butyrate engineering yeast J16 can restore the butyrate level in the intestinal cavity of patients with IBD to normal levels. The butyrate released, like previous research results,^35–37^ can regulate intestinal microbiota homeostasis, increase the abundance of probiotics, and reduce the abundance of harmful bacteria. *B. adolescentis, Bifidobacterium_adolescentis; L. johnsonii, Lactobacillus_johnsonii; L. delbrueckii, Lactobacillus_delbrueckii; L. salivarius, Lactobacillus_salivarius*. (d) The butyrate engineering yeast J16 can restore the butyrate level in the intestinal cavity of colitis mice to normal levels. The butyrate released, can inhibit the production of pro-inflammatory factors, promote the release of anti-inflammatory factors, and protect the intestinal barrier function from damage as previously reported.^38–40^ IL-6, interleukin-6; IL-1β, interleukin-1β; IL-10, interleukin-10; TNF-α, tumor necrosis factor-α; ZO1, Zona Occludens 1; MUC2, Mucin 2.

## Results

### *Construction of the butyrate biosynthetic pathway in* S. cerevisiae

**Figure 2.**
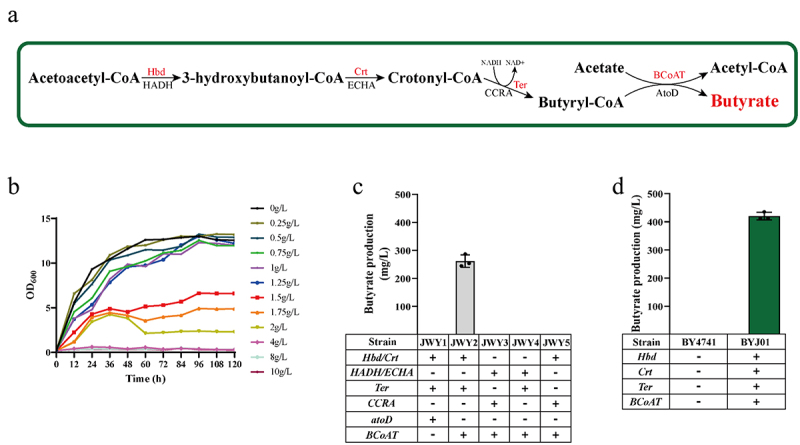
Construction of butyrate biosynthetic pathway in *S. cerevisiae*.

In order to assess the strain tolerance to butyrate, the effect of varying concentrations of butyrate on BY4741 was analyzed in YPD medium. As the concentration of butyrate was increased to 1.25 g/L (Figure 2(b)), the growth of strain BY4741 was restricted. Subsequently, higher concentrations of butyrate, such as 1.75 g/L or 2 g/L, resulted in a significant inhibition of yeast growth. Above a threshold of 4 g/L, the yeast stopped growing.

(a) Biosynthetic pathway of butyrate from acetoacetyl-CoA. The enzymes integrated into the genome of *S. cerevisiae* are written in red. Hbd; 3-hydroxybutyryl-CoA dehydrogenase from *C. beijerinckii*, HADH; 3-hydroxyacyl-CoA dehydrogenase from *F. prausnitzii*, Crt; enoyl-CoA hydratase from *C. beijerinckii*, ECHA; enoyl-CoA hydratase from *F. prausnitzii*, Ter; trans-2-enoyl-CoA reductase from *T. denticola*, CCRA; crotonyl-CoA reductase from *F. prausnitzii*, BCoAT; butyryl CoA: acetate CoA transferase from *F. prausnitzii*, atoDA; acetate CoA/acetoacetate CoA-transferase alpha subunit from *E. coli*. (b) Tolerance of yeast to different concentrations of butyrate. (c) The butyrate production of yeasts with recombinant plasmids. JWY1, yeast with pESC-His3-Hbd-Crt, pESC-Ura3-Ter and pESC-Leu2-atoD recombinant plasmids; JWY2, yeast with pESC-His3-Hbd-Crt, pESC-Ura3-Ter and pESC-Leu2-BCoAT recombinant plasmids; JWY3, yeast with pESC-His3-HADH-ECHA, pESC-Ura3-CCRA and pESC-Leu2-BCoAT recombinant plasmids; JWY4, yeast with pESC-His3-HADH-ECHA, pESC-Ura3-Ter and pESC-Leu2-BCoAT recombinant plasmids; JWY5, yeast with pESC-His3-Hbd-Crt, pESC-Ura3-CCRA and pESC-Leu2-BCoAT recombinant plasmids. (d) The butyrate production of engineered yeast BYJ01, expressing *Hbd*，*Crt*, *Ter*, and *BCoAT*.

The biosynthesis of butyrate involves several enzymatic steps starting from acetoacetyl-CoA (Figure 2(a)). Initially, acetoacetyl-CoA is converted to 3-hydroxybutanoyl-CoA through the action of 3-hydroxybutyl-CoA dehydrogenase. Subsequently, the enoyl-CoA hydratase converts 3-hydroxybutanoyl-CoA into crotonyl-CoA. The reduction of crotonyl-CoA by trans-2-enoyl-CoA reductase leads to the formation of butyryl-CoA, which is then converted to butyrate by butyryl-CoA: acetyl-CoA transferase. In this study, *C. beijerinckii Hbd* and *Crt*, which encode 3-hydroxybutyl-CoA dehydrogenase and enoyl-CoA hydratase, respectively, were selected because they have been reported to facilitate the conversion of acetoacetyl-CoA to crotonyl-CoA for n-butanol production in *S. cerevisiae* (Figure 2(a)).^45^

To achieve heterologous expression of 3-hydroxybutyl-CoA dehydrogenase and enoyl-CoA hydratase, the *Hbd* and *Crt* genes were synthesized and optimized for yeast codon preference. Subsequently, these genes were subcloned to produce the recombinant plasmid pESC-His3-Hbd-Crt (Table S1). Previous studies^46,47^ showed that trans-enoyl-CoA reductase (encoded by *Ter* and derived from *T. denticola*) and acetoacetyl-CoA transferase (encoded by *atoD* and derived from *E. coli*) can convert crotonyl-CoA to butyryl-CoA in *S. cerevisiae* and butyryl-CoA to butyrate in *E. coli*, respectively. After synthesis of *Ter* and *atoD* with codon optimization, these genes were cloned into pESC plasmids, resulting in the construction of the recombinant plasmids pESC-Ura3-Ter and pESC-Leu2d-atoD, respectively. BY4741 was then transformed with pESC-His3-Hbd-Crt, pESC-Ura3-Ter, and pESC-Leu2d-atoD to generate the recombinant strain, JWY1 (Table S1). However, butyrate was not detected in JWY1 strain (Figure 2(c)). There are no reports indicating the successful conversion of butyryl-CoA to butyrate by the *E. coli*-derived acetoacetyl-CoA transferase *atoD* in *S. cerevisiae*. *AtoD* may not have the ability to catalyze the removal of the CoA group from butyryl-CoA, thereby preventing butyrate formation in *S. cerevisiae*.

Butyrate-producing bacteria found in the digestive tract, apart from *E. col*i, possess an enzyme capable of converting butyryl-CoA to butyrate.^48^ Among these bacteria, *F. prausnitzii* is a prominent butyrate producer, constituting approximately 5% of all fecal microorganisms, and is the most abundant bacterium in the gut.^49^ To enable the heterologous expression of butyryl-CoA: acetate CoA transferase, the *BCoAT* gene encoding butyryl-CoA: acetate CoA transferase in *F. prausnitzii*, which is responsible for catalyzing the conversion of butyryl-CoA to butyrate, was artificially synthesized and optimized for yeast codon preference. The plasmid was subcloned to generate the plasmid pESC-Leu2d-BCoAT. In the recombinant strain JWY1, pESC-Leu2d-atoD was replaced with pESC-Leu2d-BCoAT, resulting in the formation of recombinant strain JWY2. Notably, the strain JWY2 exhibited a butyrate titer of 266 mg/L (Figure 2(c)). This indicated that butyrate was produced in *S. cerevisiae* through the action of *the F. prausnitzii* butyryl-CoA: acetate CoA transferase, *BCoAT*. We found that the expression of *Hbd*, *Crt*, *Ter*, and *BCoAT* in *S. cerevisiae* enabled detectable production of butyrate.

To evaluate the applicability of *F. prausnitzii*‘s butyrate synthesis system in *S. cerevisiae* for the conversion of acetoacetyl-CoA to butyrate, we utilized *HADH* (encoding 3-hydroxybutyl-CoA dehydrogenase for acetoacetyl-CoA to 3-hydroxybutanoyl-CoA conversion), *ECHA* (encoding enoyl-CoA hydratase for 3-hydroxybutanoyl-CoA to crotonyl-CoA conversion), and *CCRA* (encoding trans-2-enoyl-CoA reductase for crotonyl-CoA to butyrate conversion) as replacements for *Hbd*, *Crt*, and *Ter* or *Hbd*, *Crt*, or *Ter* individually in the JWY2 strain. Consequently, we constructed the recombinant strains JWY3, JWY4, and JWY5. However, no detectable amount of butyrate was observed during fermentation (Figure 2(c)). These results suggest that the integration of *the F. prausnitzii*‘s butyrate synthesis system into *S. cerevisiae* failed to convert acetoacetyl-CoA to butyrate.

*PGK1* and *TPI1* promoters and regulatory elements were chosen to regulate the expression of target genes such as *Hbd*, *Crt*, *Ter* and *BCoAT*. These elements were assembled into expression cassettes using molecular biological techniques. These gene cassettes were integrated into the genome of the BY4741 strain, resulting in the construction of the recombinant strain BYJ01. Butyrate production by the strain BYJ01 was 420 mg/L (Figure 2(d)).

### Increasing butyrate production by combing metabolic modules

**Figure 3.**
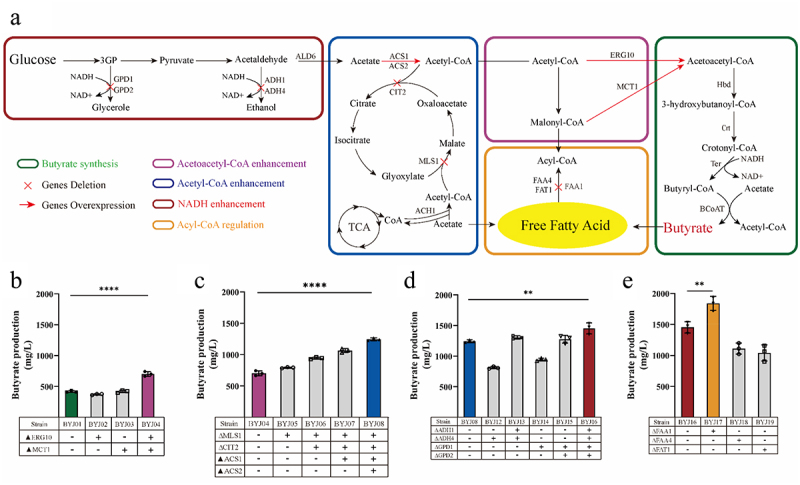
Metabolic engineering of butyrate-producing yeast.

Acetoacetyl-CoA serves as a precursor of 3-hydroxybutanoyl-CoA (Figure 3(a)). In *S. cerevisiae*, condensation of two acetyl-CoA molecules to form acetoacetyl-CoA is catalyzed by acetoacetyl-CoA thiolase, which is encoded by the endogenous *ERG10* gene.^50^ The accumulation of acetoacetyl-CoA is crucial for butyrate production. To enhance the cytosolic accumulation of acetoacetyl-CoA, we overexpressed *ERG10* of BYJ01 under control of the *TEF1* promoter and obtained the engineered strain BYJ02 (Table S1). Despite efforts to improve butyrate production, the strain BYJ02 did not show any enhancement in butyrate production (Figure 3(b)). The conversion of malonyl-CoA to acetoacetyl-CoA is catalyzed by the malonyl-CoA: ACP Transferase MCT1.^51^ We overexpressed *MCT1* in BYJ01 to improve butyrate production and constructed engineered strain BYJ03 (Table S1). However, the recombinant strain BYJ03, overexpressing *MCT1*, did not exhibit improved butyrate production (Figure 3(b)). Subsequently, we carried out simultaneous overexpression of *ERG10* and *MCT1* of BYJ01 and obtained strain BYJ04. The recombinant strain, BYJ04, demonstrated a higher capacity for butyrate production than BYJ01 (702 and 420 mg/L, respectively) (Figure 3(b)). This suggests that the simultaneous overexpression of *ERG10* and *MCT1* may accelerate the metabolism of acetoacetyl-CoA, thereby enhancing butyrate synthesis (Figure 3(a)).

(a) Biosynthetic pathway for producing butyrate. (b) The butyrate titer of the recombinant strains with acetoacetyl-CoA enhancement module. *ERG10* and *MCT1* are responsible for the synthesis of acetoacetyl-CoA. The purple column represents the strain with the highest yield. (c) The butyrate titer of the recombinant strains with acetyl-CoA enhancement module. *MLS1* and *CIT2* are responsible for the glyoxylate cycle and knockout of these two genes contributing to the accumulation of acetyl-CoA in the cytoplasm. *ACS1* and *ACS2* are responsible for the aerobic and anaerobic synthesis of acetyl-CoA, respectively. *ACH1* is responsible for CoASH transfer from succinyl-CoA to acetate. *ALD6* is required for the conversion of acetaldehyde to acetate. The blue column represents the strain with the highest yield. (d) The butyrate titer of the recombinant strains with NADH enhancement module. *ADH1* and *ADH4* participate in the synthesis of ethanol by consuming NADH. *GPD1* and *GPD2* are responsible for glycerol synthesis by consuming NADH. The red column represents the strain with the highest yield. (e) The butyrate production of the recombinant strains with acyl-CoA regulation module. *FAA1* and *FAA4* are responsible for the synthesis of long-chain fatty acids. *FAT1* is involved in the synthesis of very long-chain fatty acids and the transport of fatty acids. The orange column represents the strain with the highest yield.

Acetyl-CoA is a crucial precursor for the synthesis of acetoacetyl-CoA (Figure 3(a)). It plays a significant role in various metabolic pathways, including those involving carbohydrates, proteins, and lipids.^52^ In the cytosol, acetyl-CoA is generated from acetaldehyde by acetaldehyde dehydrogenase (ALD) and acetyl-CoA synthase (ACS).^53^ *ACS1* and *ACS2*, two genes encoding acetyl-CoA synthetases, are responsible for the conversion of acetate and CoA into acetyl-CoA.^54^ The transfer of CoA to acetate is facilitated by the acetyl-CoA hydrolase ACH1,^55^ and certain pathways can lead to the consumption of acetyl-CoA. For instance, malate synthase (encoded by *MLS1*) and citrate synthase (encoded by *CIT2*) first attach acetyl-CoA to glyoxylate (forming malate, which is further oxidized to oxaloacetate) and then to oxaloacetate (form citrate) (Figure 3(a)).^56^ Furthermore, malate synthase catalyzes the formation of β-ethylmalate from glyoxylate and butyryl-CoA,^57^ potentially leading to a decrease in butyrate production. We subsequently attempted to knock out the *MLS1* gene of BYJ04 and obtained a recombinant strain BYJ05. By deleting *MLS1*, the butyrate production by BYJ05 increased to 792 mg/L (Figure 3(c)). To further enhance the supply of acetyl-CoA to the BYJ05 strain, *CIT2* of BYJ05 was knocked out. Thus, the recombinant strain BYJ06 was obtained. BYJ06 exhibited a higher butyrate titer of 943 mg/L (Figure 3(c)). Deletion of both *MLS1* and *CIT2* effectively improved the butyrate yield. To improve acetyl-CoA biosynthesis in *S. cerevisiae*, we overexpressed *ACS1* and *ACS2* or only *ACS1* in BYJ06. Therefore, engineered strains BYJ08 and BYJ07 were obtained (Table S1). BYJ08 (overexpressing both *ACS1* and *ACS2*) showed a higher butyrate titer (1.24 g/L) than BYJ07 (overexpressing *ACS1* alone) (1.06 g/L), and both BYJ08 and BYJ07 exhibited higher butyrate production compared to BYJ06 (Figure 3(c)). By increasing the biosynthesis of acetyl-CoA in yeast, the overexpression of *ACS1* and *ACS2* improved butyrate yield. In *S. cerevisiae*, acetaldehyde is converted to acetate by aldehyde dehydrogenase (Figure 3(a)). To increase acetate supply and enhance acetyl-CoA synthesis, we overexpressed *ALD6* of BYJ08 and obtained engineered strain BYJ09. However, butyrate production was lower in BYJ09 than that in BYJ08 (Fig. S1). Interestingly, BYJ09 exhibited substantial accumulation of acetate (Fig. S2). Overexpression of *ALD6* potentially results in an excessive concentration of carbon sources in acetate rather than acetyl-CoA. Moreover, excessive acetate synthesis impaired growth of this strain (Fig. S1). To investigate the role of *ACH1* in butyrate synthesis, we deleted *ACH1* from BYJ08 to construct the recombinant strain BYJ10 (Table S1). Remarkably, butyrate production dramatically decreased to 166 mg/L compared to that of BYJ08 (Fig. S1). In addition, BYJ10 secreted a substantial amount of acetate, with an acetate titer of 11.1 g/L (Fig. S2). Growth of BYJ10 cells was also affected (Fig. S1). *ACH1* was found to be crucial for the transfer of CoA to acetate to form acetyl-CoA rather than for the hydrolysis of acetyl-CoA (Figure 3(a)).^58^ After *ACH1* deletion, CoA could not be obtained for acetyl-CoA formation.

Yeasts produce ethanol, leading to the direct transformation of a significant amount of acetaldehyde into ethanol rather than acetate (Figure 3(a)). Alcohol dehydrogenase (ADH) is responsible for catalyzing these reduction reactions. Previous studies have demonstrated that the elimination of *ADH1* and *ADH4* can effectively enhance the production of 2,3-butanediol in *S. cerevisiae*.^59^ We investigated the effects of deleting *ADH4* or both *ADH1* and *ADH4* of strain BYJ08 on butyrate production (Table S1). And recombinant strains BYJ12 and BYJ13 were obtained. The titer of butyrate in BYJ12 (with *ADH4* deleted) decreased to 0.8 g/L, lower than that of BYJ08 (Figure 3(d)). However, the deletion of both *ADH4* and *ADH1* increased butyrate production in BYJ13 (Figure 3(d)), with a titer of 1.3 g/L. Wakashima^60^ reported that CoA is required as a cofactor for the reductase enzyme Ter, which facilitates the conversion of crotonyl-CoA to butyryl-CoA (Figure 3(a)). However, NADH is consumed during the reduction of acetaldehyde to ethanol, and disruption of this reduction pathway leads to the release of NADH (Figure 3(a)). Therefore, the increase in butyrate production can be attributed to the release of NADH, which enhances the expression of *Ter*. This finding further supports the notion that trans-2-enoyl-CoA reductase, *Ter*, is a critical rate-limiting enzyme in the acetoacetyl-CoA-derived butyryl-CoA pathway (Figure 3(a)).^61^ Glycerol-3-phosphate dehydrogenase (GPD) converts glycerol-3-phosphate into glycerol and plays an essential role in lipid metabolism (Figure 3(a)).^62^ The disruption of glycerol synthesis has been shown to increase the intracellular accumulation of NADH.^59^ To increase the cytoplasmic NADH levels, we deleted *GPD1* of BYJ08 to construct engineered strain BYJ14. However, the knockout of *GPD1* alone did not significantly increase butyrate production in BYJ14 (Figure 3(d)). In order to release more NADH, we simultaneously knocked out the *GPD1* and *GPD2* genes of the BYJ08 strain and obtained a double knockout strain BYJ15. By disrupting glycerol synthesis through deletion of *GPD1* and *GPD2*, butyrate production in BYJ15 significantly improved (Figure 3(d)). We further disrupted ethanol and glycerol synthesis by knocking out *ADH1* and *ADH4*, *GPD1* and *GPD2* from BYJ08 to release NADH and obtained the recombinant strain BYJ16. Butyrate production in BYJ16 reached 1.45 g/L (Figure 3(d)).

*S. cerevisiae* naturally synthesizes fatty acids primarily composed of C16 and C18 chains.^63^ Acyl-CoA synthetases activate free fatty acids in the cytoplasm by converting them into acyl-CoA esters.^64^ In *S. cerevisiae*, six acyl-CoA synthetases are encoded by *FAA1*, *FAA2*, *FAA3*, *FAA4*, *FAT1*, and *FAT2*. Among them, the enzyme encoded by *FAA1* exhibited the highest synthetase activity.^65^ Previous studies have shown that the deletion of *FAA1* and *FAA4* leads to the release of free fatty acids.^66^ The transportation of fatty acids and the synthesis of very-long-chain fatty acyl-CoA are partially facilitated by the fatty acid transporter encoded by *FAT1*. Butyrate, a fatty acid, may be activated into an acyl-CoA ester to enter acyl-CoA synthetases (Figure 3(a)). To disrupt the acyl-CoA synthetase reaction, we individually deleted *FAA1*, *FAA4*, and *FAT1* in BYJ16, and separately obtained knockout strains BYJ17, BYJ18, and BYJ19 (Table S1). The *FAA1* knockout strain BYJ17, yielded 1.84 g/L butyrate (Figure 3(e)). However, there was no increase in butyrate production in strains BYJ18 (*FAA4* deleted) and BYJ19 (*FAT1* deleted), as the deletion of *FAA4* and *FAT1* did not affect butyrate yield (Figure 3(e)). Presumably, the knockout of *FAA1* reduced the influx of free fatty acids into fatty acid synthesis through acyl-CoA synthetases, resulting in increased butyrate secretion (Figure 3(a)).

### Performance of engineered yeasts in a complex oxygen partial pressure environment

**Figure 4.**
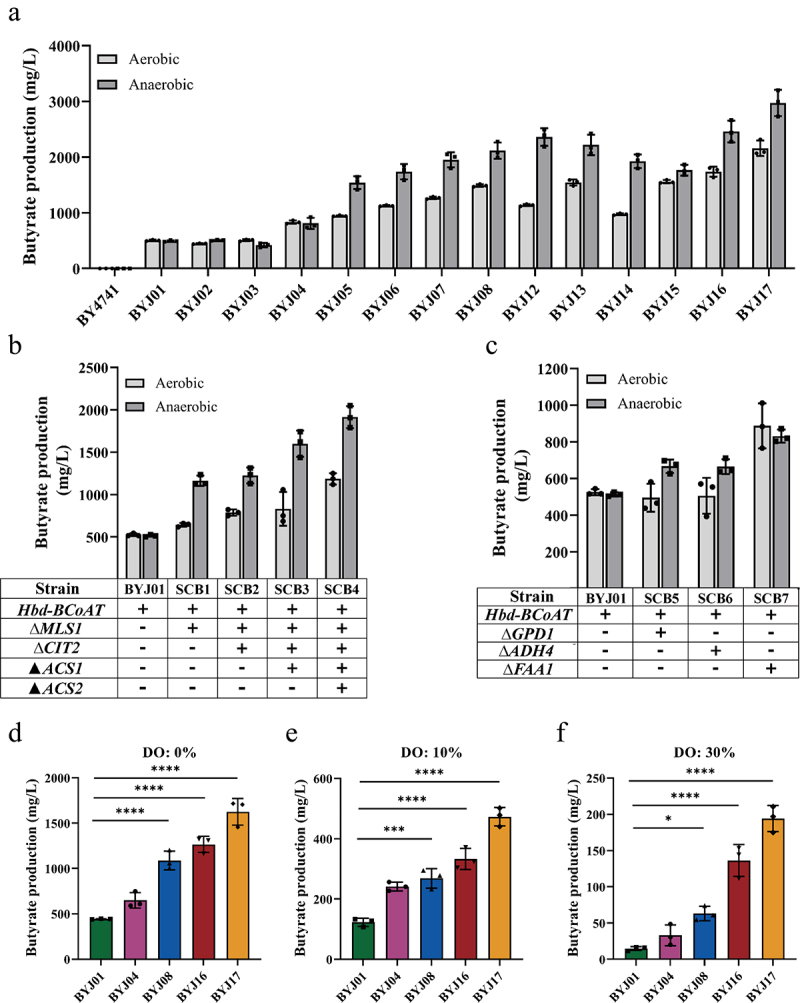
Butyrate production of engineered yeasts under different oxygen partial pressure.

Given the irregular distribution of oxygen in the intestinal tract, we conducted experiments to evaluate the performance of engineered yeasts under varying oxygen partial pressures and increased the level of the carbon source in the medium to further test the productivity of the engineered yeasts. Among the tested strains, BYJ17 exhibited the highest butyrate titer of 2.47 g/L under anaerobic conditions compared to 1.84 g/L under aerobic conditions (Figure 4(a)). Other engineered yeasts such as BYJ07, BYJ12 and BYJ16, have also demonstrated increased butyrate production under anaerobic conditions compared to that under aerobic conditions (Figure 4(a)). Hence, it can be concluded that engineered yeasts are better suited for butyrate production in anaerobic environments.

(a) In shake flasks, butyrate production of engineered yeasts under aerobic and anaerobic conditions. (b) Under aerobic and anaerobic conditions, butyrate production of the strain SCB1, SCB2, SCB3 and SCB4 (BYJ01 added with acetyl-CoA enhancement module). (c) Under aerobic and anaerobic conditions, butyrate production of the strain SCB5 and SCB6 (BYJ01 added with NADH enhancement module) and the strain SCB7 (BYJ01 added with acyl-CoA regulation module). (d – f) In the fermenter with dissolved oxygen at 0%, 10% and 30%, the butyrate production of the engineered yeasts was added with different metabolic modules, BYJ01, BYJ04, BYJ08, BYJ16 and BYJ17.

Notably, in shake flasks, the addition of the acetoacetyl-CoA enhancement module to BYJ04 did not result in strong anaerobic butyrate synthesis capabilities (Figure 4(a)). However, anaerobic butyrate synthesis in strains BYJ05, BYJ08, BYJ16, and BYJ17 was enhanced by the introduction of the acetyl-CoA enhancement module (Figure 4(a)). This suggests that certain metabolic modules enhance anaerobic butyrate synthesis in engineered yeasts. To test this hypothesis, different metabolic modules were individually applied to BYJ01 cells (Table S1). Co-overexpression of *ERG10* and *MCT1* enhances the supply of acetoacetyl-CoA. However, this did not increase butyrate production in BYJ04 under anaerobic conditions compared to that under aerobic conditions (Figure 4(a)). Meanwhile, the new strains which added acetyl-CoA enhancement module on the basis of BYJ01 were named SCB1, SCB2, SCB3, and SCB4, respectively. The new strains which equipped with NADH enhancement module on the basis of BYJ01 were named SCB5 and SCB6. The new strain equipped with acyl-CoA regulation module was named SCB7.

Conversely, knockout of *MLS1* in SCB1 resulted in increased butyrate production under anaerobic conditions compared to aerobic conditions (Figure 4(b)). Subsequently, deletion of *CIT2* in SCB1 led to increased butyrate production in SCB2 under anaerobic conditions (Figure 4(b)). By overexpressing *ACS1* in SCB2, butyrate production in SCB3 significantly increased under oxygen-limited conditions compared to that under oxygen-supplied conditions (Figure 4(b)). Further overexpression of *ACS2* in SCB3 resulted in high butyrate production under anaerobic conditions in SCB4 (Figure 4(b)). This indicated that the acetyl-CoA enhancement module significantly enhanced the anaerobic synthesis of butyrate in the engineered yeasts.

To investigate the effect of the NADH enhancement module on butyrate production in engineered yeasts under anaerobic conditions, the recombinant strains SCB5 and SCB6 were constructed by deleting *GPD1* and *ADH4*, respectively (Table S1). SCB5 and SCB6 exhibited increased butyrate production under anaerobic conditions compared to that under aerobic conditions (Figure 4(c)). To explore the role of the acyl-CoA regulation module in butyrate synthesis in the recombinant yeast, *FAA1* was deleted from BYJ01 (Table S1). However, SCB7 did not exhibit an increased butyrate titer under anaerobic conditions compared to that under aerobic conditions (Figure 4(c)).

To assess the yield of the engineered yeast strain under varying oxygen concentrations, batch fermentation was conducted using different strains equipped with diverse metabolic modules. Notably, in the absence of dissolved oxygen (DO), each strain achieved maximum butyrate production (Figure 4(d)). However, as the DO concentration increased, butyrate production gradually declined. When the amount of dissolved oxygen was increased to 10%, butyrate production was reduced (Figure 4(e)). Significant inhibition of butyrate synthesis was observed when the concentration of dissolved oxygen was increased to 30% (Figure 4(f)). These results demonstrate that the butyrate-engineered yeast can maintain optimal butyrate production even in challenging environments, particularly under hypoxic conditions.

The acetyl-CoA and NADH enhancement modules not only increased the supply of acetyl-CoA and NADH but also amplified butyrate production under anaerobic conditions. In situations with limited oxygen, improved metabolism of NADH through glycolysis and pyruvate decarboxylation, along with enhanced acetyl-CoA metabolism, play a significant role in enhancing the efficiency of butyrate synthesis in BYJ17.

### Engineered yeasts applied to the gut microbiota of patients with IBD

**Figure 5.**
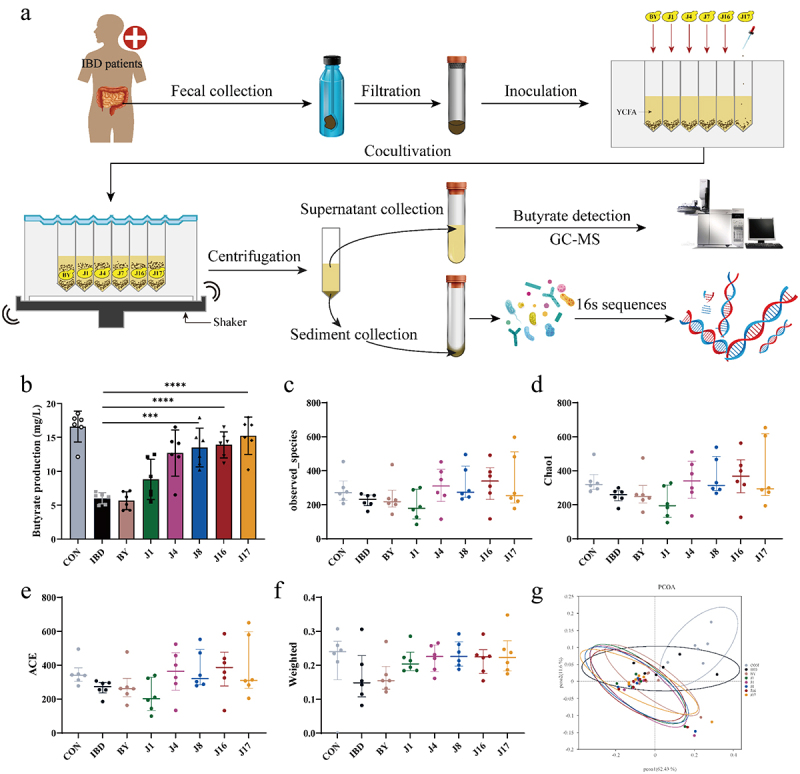
Butyrate-engineered yeasts modulate the gut microbiome homeostasis of gut microbiota from IBD patients.

Previous studies have reported that *Clostridium butyricum*, a producer of butyrate in the gut, can promote the growth of probiotics such as *Bifidobacteria* and *Lactobacilli* while inhibiting the growth of harmful bacteria.^67^ Butyrate has also been shown to improve dextran sulfate sodium (DSS) induced colitis by modulating the gut microbiota.^68^ To assess the effect of butyrate-engineered yeasts on colitis in vitro, we collected gut microbiota samples from six patients with mild-to-moderate ulcerative colitis (UC) (Figure 5(a)), and six healthy volunteers which served as the control group. Butyrate provides energy to colon cells, which consumes a large amount of oxygen, leading to a decrease in oxygen content in the intestinal environment.^69^ Considering this, we strived to minimize the exposure time of samples to the air during the sample collection process, and the entire process of patient gut microbiota inoculation and engineered yeast inoculation was completed in an anaerobic incubator. Stool samples were collected from patients and volunteers using poop cups. Phosphate Buffered Saline (PBS) was added to the samples, which were then blended using a conventional blender. After filtration, the fecal flora suspension was added to a 96-deep-well plate with Yeast Casitone Fatty Acids (YCFA) culture medium to inoculate the gut microbiota, and the engineered yeasts were added to the corresponding well positions.^70^ In order to simulate the oxygen scarce intestinal environment and unleash the potential of engineered strain to produce butyrate under anaerobic conditions, after the inoculation step was completed, we used a silicone cover to tightly seal the 96-deep-well plate to prevent oxygen from entering. The cultures were incubated on a thermostatic shaker for 24 h, followed by the separation of the cultures into supernatants and precipitation by centrifugation. The supernatant was subjected to GC – MS analysis to measure butyrate content, whereas bacterial rRNA from the precipitate was analyzed using 16S ribosomal RNA sequencing (16S rRNA-seq) (Figure 5(a)).

(a) The technical roadmap of using engineered yeasts to intervene in gut microbiota from IBD patients in vitro. GC-MS, Gas Chromatography-Mass Spectrometry. (b) The butyrate content in the culture after the different engineered yeasts intervened on the gut microbiota from IBD patients for 24 hours. (c) Observed species richness of the gut microbiota after treatments of different engineered yeasts. (d and e) The gut microbiome α-diversity analysis via Chao 1 estimator (d) and ACE index (e). (f) β-diversity analysis with weighted unifrac as a metric. (g) Principal coordinates analysis plot with Binary-Jaccard dissimilarity as a metric. CON, gut microbiota from healthy volunteers; IBD, IBD gut microbiota (gut microbiota from IBD patients); BY, IBD gut microbiota under the BY4741 (yeast chassis cells) treatment conditions; J1, IBD gut microbiota under the J1(BYJ01) treatment conditions; J4, IBD gut microbiota under the J4(BYJ04) treatment conditions; J8, IBD gut microbiota under the J8(BYJ08) treatment conditions; J16, IBD gut microbiota under the J16(BYJ16)treatment conditions; J17, IBD gut microbiota under the J17(BYJ17) treatment conditions. Data are presented as means ± SEM (*n* = 6). Statistical analysis was performed using one-way ANOVA with Tukey’s multiple comparison test. *p < .05, **p < .01, ***p < .001 and ****p < .0001.

The butyrate content in the cultures increased with the enhancement of butyrate synthesis through the addition of different metabolic modules to butyrate-engineered yeasts (from J1 to J17) (Figure 5(b)). Among these strains, J17 exhibited the highest butyrate production. In terms of bacterial richness, as indicated by the observed species, rarefaction curve, rank abundance, and gut microbiome α-diversity measured by Chao 1 and the abundance Coverage-based Estimator (ACE), the J16 group showed significant differences compared to the IBD group

(Figure 5(c–e) and Figure S3). However, yeast chassis cells BY and engineered strains J1, J4, J8 and J17 did not significantly improve the microbial community structure in patients with IBD. This was attributed to the fact that the amount of butyrate released by engineered strain needs to be controlled within a certain range to maximize the improvement effect of engineered strain on gut microbiota (Figure 5(b)).

By comparing the gut microbiota diversity of each treatment group using the β-diversity index (Figure 5(f)), we observed a significant reduction in diversity in the IBD group. However, intervention with butyrate-producing engineered yeasts, such as J4, J8, J16, and J17, could reverse this change (Figure 5(f)), suggesting that the secretion of butyrate by these engineered yeasts could improve the composition of the gut microbiota in patients with IBD. Additionally, principal coordinate analysis of the gut microbiome using Binary-Jaccard dissimilarity as a metric (Figure 5(g)) revealed distinct differences between the gut microbiota profiles of the J4, J8, J16, and J17 groups and those of the IBD group. This indicates that the engineered yeasts significantly altered the structure of the gut microbiota by producing butyrate.

**Figure 6.**
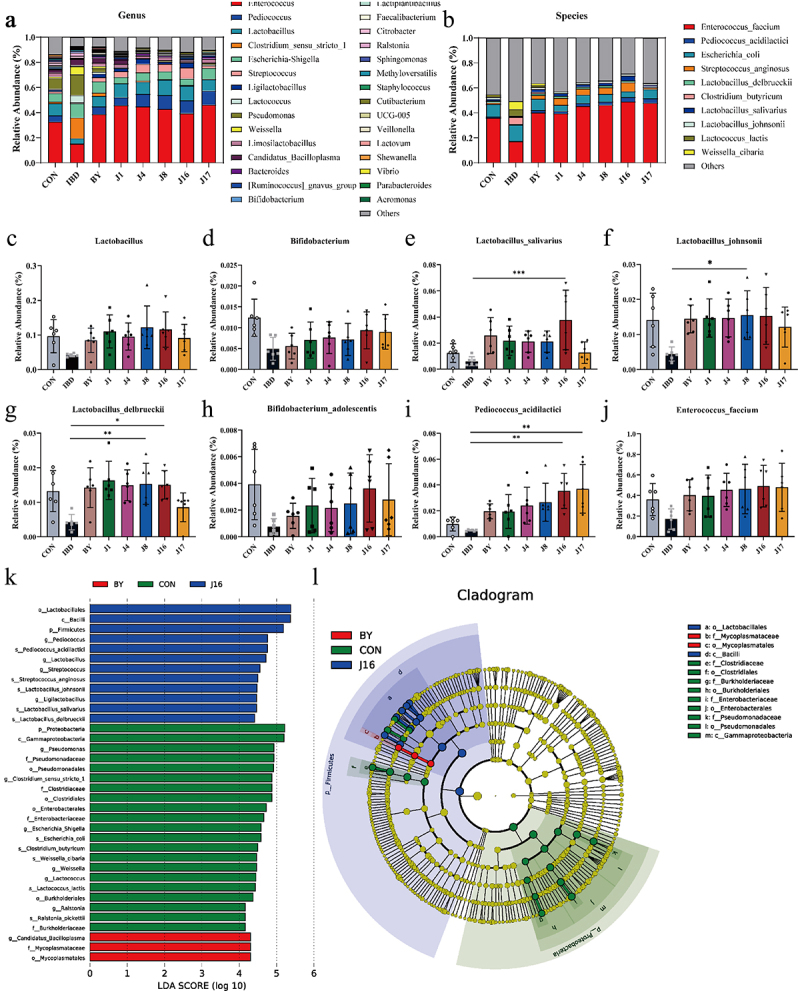
Effect of engineered yeasts on the species composition of the IBD gut microbiota.

Upon closer investigation at the genus level (Figure 6(a) and S4), it was revealed that engineered strain that release specific amounts of butyrate, such as J8 and J16, had a profound effect on the abundance of beneficial bacteria, such as *Lactobacillus* Compared to the IBD group (Figure 6(c) and Fig. S4) within the gut microbiota of individuals diagnosed with IBD. Compared with other engineered strains treatment groups, the J8 and J16 groups slightly increased the abundance of *Lactobacillus*. Similar positive effects were observed for *Enterococcus* and *Pediococcus* (Fig. S4). *Lactobacillus* is a well-known probiotic that alleviates intestinal inflammation.^71^ Among the yeast strains tested, J8 demonstrated the most pronounced effect on augmenting the abundance of *Lactobacillus*. However, the effectiveness started to decline after intervention by J16 and J17 (Figure 6(c)). The abundance of *Lactobacillus* treated with J17 was comparable to that treated with J4, likely because of the high concentration of butyrate secreted by J17. Additionally, the investigation revealed that butyrate-engineered yeasts J16 effectively increased the relative abundance of *Bifidobacterium* in the gut microbiota of patients with IBD compared with IBD group (Figure 6(d)). Moreover, the J16 group showed a slight increase in the abundance of *Bifidobacterium* compared to other engineered strains treatment groups. *Bifidobacterium*, a crucial member of the gut microbiota, plays a significant role in maintaining intestinal health and reducing gastrointestinal disorders.^72,73^ However, the impact of J17 was not as significant as that of J16 (Figure 6(d)), possibly because of the excessive production of butyrate by J17 (Figure 5(b)). Furthermore, examination at the species level (Figure 6(b)) revealed that treatment with butyrate-engineered yeasts (J8, J16, and J17) significantly increased the relative abundances of *Lactobacillus salivarius* (Figure 6(e)), *Lactobacillus johnsonii* (Figure 6(f)), *Lactobacillus delbrueckii* (Figure 6(g)), *Bifidobacterium adolescentis* (Figure 6(h)), *Pediococcus acidilactici* (Figure 6(i)) and *Enterococcus faecium* (Figure 6(j)). *L. salivarius* alleviated inflammation and restored a balanced environment in a DSS-induced colitis model.^74^ *L. johnsonii* has shown efficacy in reducing infection by the diplomonad *Giardia intestinalis* in gerbils and in inhibiting *C. perfringens* colonization in chickens.^75,76^ Studies have indicated that the oral administration of *L. delbrueckii* regulates mucosal and systemic immune responses in mice with colitis.^77^ *B. adolescentis* promoted Th17 cell accumulation, thereby contributing to the maintenance of intestinal homeostasis.^78^ *P. acidilactici* has demonstrated efficacy in reducing clinical and intestinal allergic reactions in allergic mice^79^ and in alleviating inflammation in mice with colitis.^80^ *E. faecium* is widely used as a probiotic for pharmaceutical and animal nutritional applications.^81^ The relative abundances of other probiotics decreased to varying degrees after J17 treatment, including *L. salivarius*, *L. johnsonii*, *L. delbrueckii* and *B. adolescentis* (Figure 6(e–h)). The therapeutic effect of J16 was the most prominent, as it maximized the relative abundance of probiotics, such as *L. salivarius* and *B. adolescentis* (Figure 6(g,h)), indicating that moderate supplementation of butyrate from engineered strains is necessary to achieve optimal therapeutic outcomes. Moreover, treatment with J4, J8, J16, and J17 markedly reduced the relative abundance of *Candidatus Bacilloplasma*, which is known to be enriched in diseased shrimp (Fig. S5).^82^ Linear discriminant analysis effect size (LEfSe) identified the dominant microbiota and biomarkers at a threshold of 4 (Figure 6(k,l)). At the genus level, the dominant microbiota in J16 group were *Pediococcus*, *Lactobacillus*, *Streptococcus*, and *Ligilactobacillus*. The dominant microbiota in the BY group was *Candidatus Bacilloplasma*. The dominant microbiota in the CON (Control) group (gut microbiota from healthy volunteers) were *Pseudomonas*, *Clostridium sensu stricto 1*, *Escherichia-Shigella*, *Weissella*, *Lactococcus*, and *Ralstonia*. At the species level, the dominant microbiota in the J16 group was *Pediococcus acidilactici*, *Streptococcus anginosus*, *Lactobacillus johnsonii*, *Lactobacillus salivarius*, and *Lactobacillus delbrueckii*. The dominant microbiota in the CON group included *Clostridium butyricum*, *Weissella cibaria*, *Lactococcus lactis*, and *Ralstonia pickettii*. In the ternary diagram, when comparing the dominant microorganisms at the genus level among the three groups: J16, BY, and CON, it become evident that *Streptococcus*, *Pediococcus*, *Ligilactobacillus*, *Lactobacillus*, and *Enterococcus* were relatively abundant in the J16 group (Fig. S6A). At the species level, the densities of *Streptococcus anginosus*, *Pediococcus acidilactici*, *Lactobacillus salivarius*, *Lactobacillus delbrueckii*, *Lactobacillus johnsonii*, and *Enterococcus faecium* were higher in the J16 group than that in the BY and CON groups (Fig. S6B).

(a) Relative abundance at the genus level. (b) Relative abundance at the species level. (c) Relative abundance of *Lactobacillus* at the genus level. (d) Relative abundance of *Bifidobacterium* at the genus level. (e) Relative abundance of *Lactobacillus_salivarius* at the species level. (f) Relative abundance of *Lactobacillus_johnsonii* at the species level. (g) Relative abundance of *Lactobacillus_delbrueckii* at the species level. (h) Relative abundance of *Bifidobacterium_adolescenti*s at the species level. (i) Relative abundance of *Pediococcus_acidilactici* at the species level. (J) Relative abundance of *Enterococcus_faecium* at the species level. (k) Differentially enriched intestinal microbiota in all groups by linear discriminant analysis (LDA). An LDA score > 4 represents a higher abundance in the group than that in other groups. (l) Cladogram based on linear discriminant analysis effect size (LEfSe) analysis. Data are presented as means ± SEM (*n* = 6). Statistical analysis was performed using one-way ANOVA with Tukey’s multiple comparison test. *p < .05, **p < .01, ***p < .001 and ****p < .0001.

In conclusion, butyrate-engineered yeasts (J8, J16, and J17) regulated homeostasis of the gut microbiome in patients with IBD by producing butyrate, increasing the relative abundance of probiotics such as *Lactobacillus* and *Bifidobacteria*, and reducing the presence of harmful bacteria. However, owing to differences in butyrate production, the therapeutic effects of these engineered yeasts differed. Among these, J16 secreted an optimal amount of butyrate, leading to the most significant therapeutic effect on the gut microbiota of individuals with mild-to-moderate UC. This is reflected in its ability to maximize the relative abundance of most probiotics and effectively modulate gut microbiome homeostasis.

### Therapeutic efficacy of engineered yeasts in TNBS-induced IBD mice model

**Figure 7.**
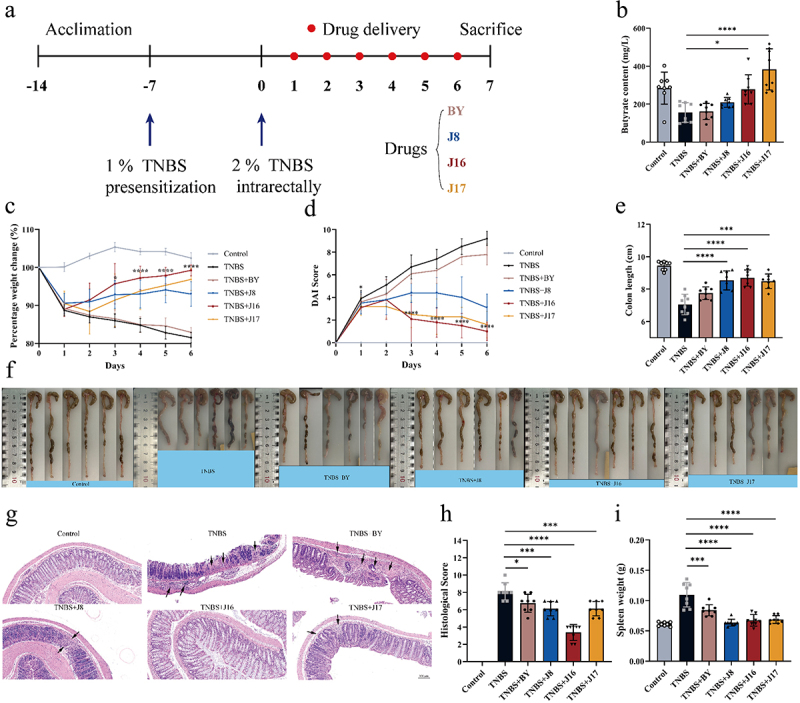
Therapeutic efficacy of engineered yeasts against TNBS-induced mouse colitis model.

Based on the successful in vitro regulation of intestinal bacteria by the engineered yeasts, their therapeutic effectiveness was further investigated in vivo by treating TNBS-induced C57BL/6 mice with colitis. The experimental design involved administering a 2 weight % (wt %) TNBS enema to C57BL/6 mice on day 0 (Figure 7(a)). Mice were divided into six groups: control, model (TNBS), BY treatment (TNBS+BY), J8 treatment (TNBS+J8), J16 treatment (TNBS+J16), and J17 treatment (TNBS+J17). Except for the control and model groups, mice in the remaining groups were orally administered various engineered yeasts (BY, J8, J16, and J17) daily until euthanasia on day 7 (bacterial dose: 1 × 10^8^ colony-forming units [CFU]). Butyrate levels in the colonic contents of mice were measured to evaluate the ability of the engineered yeast to secrete butyrate into the intestine. The therapeutic efficacy of the engineered yeast was assessed by monitoring the changes in body weight, disease activity index (DAI), colon length, histological analysis of colon sections, histological scores, and spleen weight.

(a) Experimental scheme for TNBS-induced colitis and engineered yeasts (BY, J8, J16 and J17) administration. (b) Butyrate content in the colon lumen of mice in each group. (c–e) Daily body weight, (d) Daily DAI scores and (e) Colon length of mice in each group via rectal enema were monitored on day 7. (f) Colon tissue images of mice in each group. (g) Representative images of colon sections of indicated groups via oral gavage were stained with H&E. Scale bars, 100 μm. (h) Colonic damage scores of mice in each group. (i) Spleen weight of mice in each group. Data are means ± SEM (*n* = 6 to 8); *p < .05, **p < .01, ***p < .001 and ****p < .0001 by one-way ANOVA with Tukey’s multiple comparison test.

Consistent with the in vitro experimental results, the butyrate content in the intestinal cavity of mice varied among the different treatment groups (Figure 7(b)). The mice in the BY treatment group had the lowest butyrate content because of the limited ability of the yeast strain BY4741 to synthesize butyrate. In contrast, the J17 treatment group exhibited the highest intestinal butyrate content because J17 harbored all the metabolic modules. However, the butyrate concentration in the intestinal cavity of mice from the J17 treatment group exceeded that of the control group, which could potentially have an adverse therapeutic effect on colitis in mice. Notably, supplementation with butyrate through J16 (slightly weaker butyrate production compared to J17) restored the luminal butyrate content to normal levels in mice with colitis (Figure 7(b)). Thus, the amount of butyrate provided by J16 was optimal for the treatment of colitis in mice.

While BY did not improve the severity of TNBS-induced acute colitis in mice, mice in the TNBS+J8, TNBS+J16, and TNBS+J17 groups showed increased body weight (Figure 7(c)), decreased disease activity index (DAI) (Figure 7(d)), longer colon length (Figure 7(e,f)), and reduced spleen weight (Figure 7(i)) compared to those in the TNBS group. The therapeutic effect of the engineered yeast on mice with colitis primarily stems from its ability to secrete substantial amounts of butyrate, which is known for its potent anti-inflammatory properties.^7^ Among the engineered yeasts, J16 exhibited the most prominent therapeutic effect in mice, whereas the therapeutic effect of J17 was less pronounced than that of J16. Histological analysis revealed significant crypt defects and mucosal muscle layer infiltration in the colons of mice with colitis in the TNBS group. However, treatment with engineered yeasts (J8, J16, and J17) led to improved colon histology in mice with colitis, with reduced inflammatory cell infiltration and increased crypts. Notably, J16 exhibits remarkable therapeutic effects (Figure 7(g,h)).

It is important to note that a higher secretion of butyrate from engineered yeast did not necessarily yield better results. Although J17 produced the highest butyrate content in the intestinal cavity of mice, its therapeutic effect on mice with colitis was less pronounced than that of J16, as evidenced by lower body weight, higher DAI scores, shorter colon length, heavier spleen weight, and higher histological scores (Figure 7(b–i)). These findings indicate that the amount of butyrate secreted by J16, rather than J17, fulfilled the therapeutic requirements of mice with colitis.

**Figure 8.**
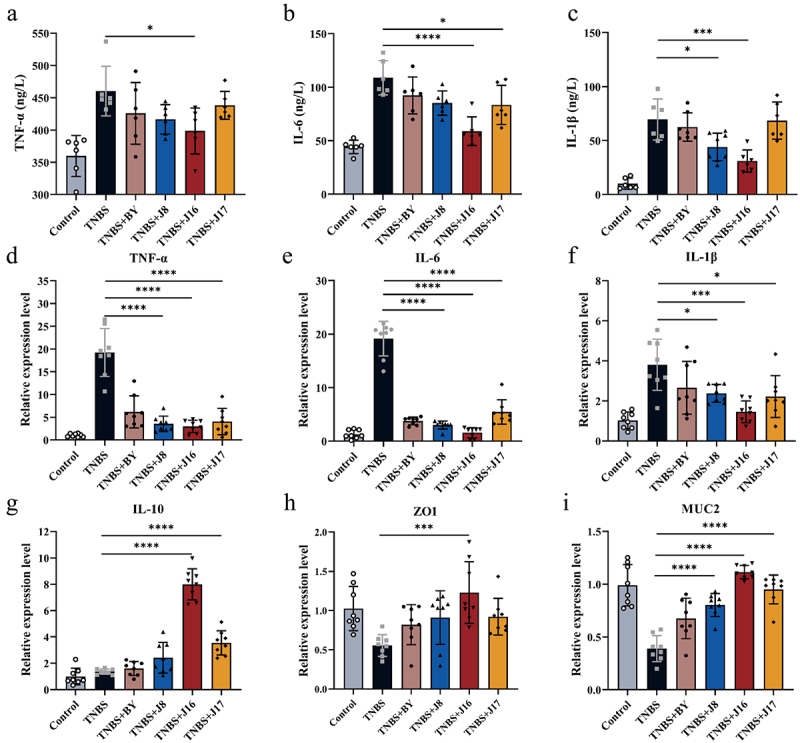
Therapeutic mechanisms of the engineered butyrate on IBD.

Owing to its remarkable anti-inflammatory properties, butyrate is considered a potential therapeutic agent for IBD.^7^ To further investigate the therapeutic mechanisms of the butyrate-engineered yeasts, we analyzed the levels of pro-inflammatory mediators in the serum. The TNBS group exhibited higher levels of tumor necrosis factor-α (TNF-α), interleukin-6 (IL-6), and interleukin-1β (IL-1β) compared to the control group. However, engineered yeasts J8, J16, and J17 effectively reduced the levels of pro-inflammatory cytokines TNF-α, IL-6, and IL-1β when compared to the TNBS group (Figure 8(a–c)). Among the engineered yeasts, J16 demonstrated the most pronounced anti-inflammatory effect, likely because of the appropriate amount of butyrate secreted to treat TNBS-induced colitis in mice (Figure 8(a–c)). The therapeutic effect of J17 was less favorable than that of J16, possibly because of excessive butyrate secretion.

(a–c) The content of TNF-α, IL-6 and IL-1β in the serum of mice was measured. The serum was measured using the SenBeiJia Biological Technology mouse (TNF-α, IL-6 and IL-1β) ELISA Kit. (d–g) Relative expression level of TNF-α, IL-6, IL-1β and IL-10 in the colon. (H to I) The relative expression level of ZO1 and MUC2 in the colon. Data are means ± SEM (*n* = 6 to 8); *p < .05, **p < .01, ***p < .001 and ****p < .0001 by one-way ANOVA with Tukey’s multiple comparison test.

To further validate the therapeutic effect of engineered yeasts, we measured the mRNA expression of pro-inflammatory and anti-inflammatory cytokines in the colonic mucosa, including TNF-α, IL-6, IL-1β, and IL-10, which play crucial roles in IBD pathologies (Figure 8(d-g)). As expected, the engineered yeast significantly reduced the levels of pro-inflammatory cytokines and increased those of anti-inflammatory cytokines. Notably, J16 exhibited the most significant treatment effect, with lower levels of inflammatory factors, such as IL-6, and higher levels of anti-inflammatory factors, such as IL-10. This indicated that J16 possesses a prominent anti-inflammatory effect. Conversely, the anti-inflammatory effects of J17 were less pronounced than those of J16.

Butyrate supports the function and integrity of the intestinal barrier by regulating tight junctions and mucus production.^8^ To confirm the protective effect of the butyrate-engineered yeast on the intestinal barrier, we measured the relative expression of colonic intestinal barrier indicators. Consistent with the anti-inflammatory effect, the engineered yeast effectively preserved the integrity of the intestinal barrier, preventing immune cell-mediated destruction of the tight junction proteins ZO1 and mucin MUC2 through butyrate transport. Among the engineered yeasts, J16 demonstrated optimal protection, with the highest expression levels of ZO1 and MUC2 (Figure 8(h,i)). The effectiveness of J17 in maintaining intestinal barrier integrity was not as significant as that of J16. These findings highlight the importance of controlling butyrate dosage in colitis treatment. By leveraging synthetic biology, engineered yeasts can produce appropriate amounts of butyrate to maximize their therapeutic effects on colitis.

## Discussion

In recent years, significant advancements have been made in the treatment of IBD. The introduction of biological therapies, including antitumor necrosis factor agents and integrin inhibitors, has revolutionized the management of IBD. These therapies have been proven to be highly effective in inducing and maintaining remission.^83^ Despite these notable achievements, challenges persist in the treatment of IBD, such as the complex and heterogeneous nature of the disease, limited treatment options, and side effects of medication.

Short-chain fatty acids are produced through the fermentation of dietary fiber by beneficial bacteria in the cecum and colon. They play crucial physiological roles in the intestine, providing energy to intestinal cells, maintaining the integrity of the intestinal mucosal barrier, and regulating immune responses.^84^ Butyrate, a specific short-chain fatty acid, has beneficial effects on intestinal health. It promotes the well-being of intestinal cells, enhances the integrity of the intestinal barrier, and exhibits anti-inflammatory properties.^85^ Intestinal butyrate levels are often reduced in patients with IBD.^32^

Given the limitations associated with the direct administration of butyrate and other supplementation methods, we developed a novel approach for intestinal treatment using butyrate-engineered yeasts. Our strategy involved identifying the key genes responsible for butyrate production in various hosts, optimizing them for yeast codon preference, and synthesizing these gene sequences. These genes were expressed in yeast cells using plasmids, and exogenous synthetic genes suitable for butyrate production in yeast were selected. To assemble the expression cassettes, we used molecular biology techniques and incorporated suitable promoters and regulatory elements. These expression cassettes were inserted into the genome of *S. cerevisiae*, and subsequent screening identified strains capable of producing butyrate. However, recognizing that butyrate production by these engineered strains was insufficient to meet clinical therapeutic requirements, we employed four metabolic engineering approaches to further optimize their performance. First, given the direct involvement of acetoacetyl-CoA in butyrate production, we introduced an acetoacetyl-CoA enhancement module to bolster the substrate supply for butyrate synthesis (Figure 3(a)). Next, an acetyl-CoA enhancement module was incorporated to augment the availability of acetyl-CoA, which plays direct and indirect roles in acetoacetyl-CoA and butyrate synthesis, respectively (Figure 3(a)). Furthermore, because the butyrate synthesis pathway relies on an adequate supply of NADH and necessitates carbon cycle concentration, we introduced an NADH enhancement module (Figure 3(a)). Finally, to prevent butyrate from being consumed during the synthesis of long-chain fatty acids in yeast, we implemented an acyl-CoA regulation module to increase butyrate production (Figure 3(a)). Our final butyrate-engineered yeast strains successfully produced 1.8 g/L of butyrate (Figure 3(e)). Additionally, by reinforcing the supply of acetyl-CoA and NADH, we enhanced the performance of the butyrate-engineered yeast strains under anaerobic conditions. This enabled the engineered yeast to sustain a certain level of butyrate production in the intestinal environment, thereby ensuring a therapeutic effect.

We then validated the efficacy of butyrate-engineered yeast. In in vitro experiments, we employed engineered yeasts to intervene with the gut microbiota from patients with IBD and observed that the butyrate production of each engineered strain remained stable (Figure 5(b)). Moreover, the high-yield engineered strains (J8, J16, and J17) exhibited favorable regulatory effects on the gut microbiota of patients with IBD, notably increasing microbial diversity. These engineered strains demonstrated a significant increase in the abundance of beneficial probiotics, including *Lactobacillus*, *Bifidobacteria* at the genus level (Figure 6(c,d)), and *Pediococcus acidilactici*, *Lactobacillus delbrueckii*, *Lactobacillus salivarius*, *Lactobacillus johnsonii*, and *Enterococcus faecium* at the species level (Figure 6(e–j)). Simultaneously, through the release of butyrate, the high-butyrate-producing engineered strains effectively reduced the abundance of harmful bacteria, such as *Candidatus Bacilloplasma* (Fig. S5). However, we observed that strain J16, which had the second highest butyrate yield, exhibited superior therapeutic effects on the gut microbiota of patients with IBD compared with that of strain J17, which had the highest butyrate yield. Under treatment with the J16 strain, populations of probiotics such as *Lactobacillus* reached their highest levels. Therefore, we speculated that the amount of butyrate released by the engineered strain should be controlled within a specific range to ensure optimal therapeutic outcomes.

To test this hypothesis, we used a mouse model of colitis to evaluate the therapeutic effects of the engineered yeasts with different levels of butyrate production. Butyrate was detected in the colonic contents of mice to confirm the performance of the engineered yeasts in the gut environment (Figure 7(b)). Remarkably, all butyrate-engineered strains effectively treated colitis. However, the therapeutic effect did not increase linearly with an increase in butyrate content. Surprisingly, strain J16 showed the most significant therapeutic effect in mice with colitis. This effect was evident through weight restoration, maintenance of colonic structure, anti-inflammatory properties, and protection of the intestinal barrier. Our experiments demonstrate that butyrate-engineered yeasts possess therapeutic potential in colitis treatment. These findings highlight the importance of controlling butyrate production in engineered strains to achieve optimal therapeutic outcomes.

At the same time, it is important to note that the butyrate-engineered strain Inhibited the occurrence of intestinal inflammation through multiple factors. It depends on the butyrate produced by the engineered strain. Butyrate has been extensively confirmed to be widely involved in host immune regulation and has excellent anti-inflammatory properties.^86–88^ On the one hand, butyrate, as a competitive HDACi, inhibits HDAC activity in intestinal epithelial cells and immune cells.^38,39^ Butyrate, as HDACi, downregulates NF-κB activity and inhibits intestinal inflammatory response.^40,41^ On the other hand, butyrate inhibits intestinal inflammation, regulates immune response, and improves intestinal barrier by mediating the activation of G protein coupled receptors on the surface of intestinal epithelial cells, such as GPR41, GPR43 and GPR109A.^42–44^ There is an interactive symbiotic effect between butyrate producing bacteria and beneficial bacteria like *Lactobacillus* and *Bifidobacterium* in the intestine, which can promote the growth of beneficial gut microbiota.^36,37^ Our research indicated that engineered strain can increase the abundance of *Lactobacillus* and *Bifidobacterium*. The proliferation and growth of *Lactobacillus* and *Bifidobacterium* in the intestine can reduce the number of harmful bacteria, thereby regulating the microecological balance of the intestinal microbiota, which is crucial for protecting the intestinal barrier, reducing intestinal inflammatory reactions, and maintaining intestinal homeostasis.^89–91^

In addition to its established therapeutic effects on IBD, butyrate has shown promise for the treatment of colorectal cancer, metabolic disorders, and neurological disorders. Butyrate exhibits anticancer properties and inhibits the growth of colorectal cancer cells.^34^ In the context of metabolic disorders, butyrate has beneficial effects in conditions such as obesity, insulin resistance, and type 2 diabetes.^92^ It improves insulin sensitivity, regulates glucose metabolism, and aids in weight management.^93^ Furthermore, the regulatory effects of butyrate on neurological disorders through the brain-gut axis manifest in its ability to modulate the gut microbiota, regulate neurotransmitter levels, reduce inflammation, and support intestinal barrier function.^93^ Notably, the optimal therapeutic dose of butyrate varies depending on the specific disease and the individual patient. Single-concentration supplementation with butyrate failed to meet the diverse needs of all patients. However, through synthetic biology, we can achieve heterologous expression of butyrate using *S. cerevisiae*. By leveraging the metabolic network of probiotics, the corresponding metabolic modules can be regulated based on different indications and application environments. Engineered yeast can maximize therapeutic efficacy by secreting an appropriate concentration of butyrate at the site of the lesion. For instance, some patients have greater requirements for butyrate, and the supply of precursor substances can be maximized by regulating the metabolic modules of the precursor substances and enhancing butyrate production in engineered strains.

In addition to the direct regulation of probiotic butyrate secretion through metabolic networks, synthetic biology has the potential to enable probiotics to deliver therapeutic substances based on disease-related signals.^94–96^ For instance, Benjamin^27^ introduced a self-tunable P2Y2-RROP1 gene circuit into *S. cerevisiae*, enabling the engineered yeast to respond specifically to extracellular adenosine triphosphate (eATP) produced in the inflamed gut. This response triggers the secretion of apyrase, which alleviates intestinal inflammation by consuming eATP. Numerous studies have indicated a significant reduction in the butyrate content within the intestinal lumen of patients with conditions related to butyrate metabolism disorders, including gut inflammation, colorectal cancer, lipid metabolism disorders, and psychiatric disorders influenced by the brain-gut axis.^10,32,97^ Thus, butyrate-engineered yeast can autonomously adjust the amount of butyrate released by sensing a decrease in the butyrate content in the environment. For instance, Bai^98^ developed a high-throughput biosensor for *E. coli* that responded to intracellular butyrate concentrations. Furthermore, a recent study by Dang^99^ summarized synthetic bacterial therapies for gut diseases, highlighting the potential for the targeted and controlled release of therapeutic drug molecules by sensing physiological signals associated with intestinal diseases. To implement a butyrate-sensing system, it can be introduced into butyrate-engineered yeast strains, such as J17. This can be accomplished by incorporating a specific sensor protein or gene that responds to the butyrate levels. Subsequently, a genetic circuit can be established to integrate the sensing mechanism and control the expression of the genes responsible for butyrate production. This circuit activates or enhances the expression of these genes when the concentration of sensed butyrate is low. Promoters of butyrate-producing genes can be modified to respond to the regulatory circuit, ensuring that the expression of these genes is controlled by the regulatory system and synchronized with sensed butyrate levels. Finally, the engineered strain could produce butyrate in response to various environmental butyrate levels.

## Conclusions

In summary, this study used a synthetic biological method to realize butyrate synthesis in the *S. cerevisiae* strain and regulate related metabolic modules to continuously adjust the butyrate production of engineered yeasts to achieve the best therapeutic effect on colitis in vivo and in vitro. This study is expected to provide new insights and references for the targeted and better treatment of related diseases.

## Materials and methods

### Strains and media

*S. cerevisiae* BY4741 (MATa, his3, leu2, met15, and ura3) was used to construct a recombinant strain because its genome was used to construct all linear DNA for genome editing. Yeast strains were cultured at 30°C with shaking (220 rpm) in yeast extract peptone dextrose (YPD) medium [10 g/L yeast extract, 20 g/L Bactopeptone (Difco Laboratories), 20 g/L glucose. The level of glucose was increased to 30 g/L to test the productivity of the strain at different oxygen partial pressures] or synthetic dropout (SD) medium (1.7 g/L yeast nitrogen base, 5 g/L ammonium sulfate, and 20 g/L glucose) with supplemental amino acids (Difco Laboratories). Additionally, 2% (w/v) agar was added as required. *Escherichia coli* DH5ɑ was used for maintaining and propagating recombinant plasmids and was cultured at 30°C with shaking (220 rpm) in Luria-Bertani medium (10 g/L tryptone, 5 g/L yeast extract, and 10 g/L NaCl). Ampicillin was used at a final concentration of 100 g/L, when necessary.

### Yeast resistance to butyrate

The tolerance of yeast to butyrate was evaluated by adding different concentrations of butyrate purchased from Macklin to the YPD medium and monitoring the growth of the strains. The growth of the strains was analyzed every 12 h by measuring the optical density of the cultures at 600 nm (OD_600_).

### Fermentation conditions

To strengthen their viability, the yeast strains were cultivated twice in the YPD medium for 16 h at 30°C. For induced fermentation, 50 mL of SD-HIS-LEU-URA medium (with histidine, leucine, and uracil removed) in a 250 mL shake flask was used as the fermentation medium. The yeast cell biomass reached an OD_600_ of 2 in YPD medium. Yeast cells were centrifuged at 2200 × g for 5 min and washed with 10 mL distilled water. The cells were then inoculated in SD-HIS-LEU-URA fermentation medium at 30°C with shaking (220 rpm) for approximately 7 days.

For aerobic fermentation, 50 mL of YPD medium with 40 g/L glucose in a 250 mL shake flask was used as the fermentation medium. Sealed the bottle mouth with breathable sealing film to ensure air exchange. After cultivation in YPD medium, yeast cells were inoculated in YPD medium at an initial OD_600_ of 0.2. The strains were grown at 30°C with agitation at 220 rpm for 7 days. For anaerobic fermentation, 50 mL of YPD medium with 40 g/L glucose in a 250 mL shake flask was used as the fermentation medium. After cultivation in YPD medium, yeast cells were inoculated in YPD medium at an initial OD_600_ of 0.2. Nitrogen was injected into the flask with the YPD medium to remove residual oxygen. The flasks were sealed with Parafilm to avoid the entry of oxygen from outside. At the same time, we covered the outside of the flask with two layers of tin foil to completely isolate the contact between the incubator and the external air. The strains were grown at 30°C with agitation at 220 rpm for 7 days.

Batch fermentation was performed in a quadruple glass fermenter (Bilbao, China). The strains were activated twice and inoculated into 1 L of YPD medium in a fermenter with an initial OD_600_ of 5. The temperature was controlled at 30°C in a 1.5 L fermentation volume. The pH was maintained at 5.5 by the dropwise addition of 5 N H_2_SO_4_ or 5 N NaOH. The dissolved oxygen in the reaction system was maintained at 0%, 10%, and 30% by controlling the airflow rate. The fermentation was performed at an agitation speed of 300 rpm for 4 days.

### Metabolite analysis by GC – MS

For pretreatment of the yeast culture medium, refer to the previously described method. To acidify the collected supernatant test samples, 2 mL of supernatant culture medium was aliquoted into a 5 mL polyethene centrifuge tube, and 0.4 mL of 50% sulfuric acid and 2.6 mL diethyl ether was added into the supernatant. The mixture was incubated in a shaker at 30°C with shaking (200 rpm) for 45 min, and then centrifuged at 3000 rpm for 5 min. The supernatant was removed and placed in another sterile centrifuge tube, and anhydrous calcium chloride was added for dehydration and filtered through 0.22 μm nylon filters. Calcium chloride was used to remove water, and the supernatant was used for GC – MS detection.

For the GC – MS process, a Column Agilent 123–7032 DB-WAX was used. The column temperature started at 60°C, which was held for 2 min, ramped up to 220°C at 10°C/min and then held at this temperature for 20 min. Helium was used as the carrier gas. The flow rate was 1 mL/min, the split ratio was 20:1, the injection volume was 2 μL, and the starting temperature of the injection port was 250°C. MS conditions: an EI ion source, 70 eV; Ion source temperature, 230°C; quadrupole temperature, 150°C; solvent delay time, 2 min; scan mass range, m/z 20–150.

### Patient stool sample collection

Stool samples were collected from six healthy volunteers and six patients with mild-to-moderate ulcerative colitis (UC) referred to the Tianjin Medical University General Hospital from November 2022 to December 2022. Patients were diagnosed with mild-to-moderate UC based on clinical, histological, radiological, and colonoscopic criteria. The inclusion criteria were as follows: (1) subjects voluntarily donated their own stools and signed an informed consent form; (2) subjects aged 31–59 years, both sexes were included; (3) subjects met the diagnostic criteria for mild-to-moderate UC and a Mayo score of 4–10. The exclusion criteria were as follows: (1) subjects who were pregnant or unable to provide informed consent; (2) subjects who had suffered from severe immunodeficiency in the previous 6 months; (3) subjects who had taken antibiotics or probiotics within the previous 6 weeks; (4) subjects who had taken blood pressure medicines within the previous 6 weeks; (5) subjects who had suffered from underlying systemic diseases in the previous 6 months; and (6) subjects who had suffered severe UC (Mayo score > 10) in the previous 6 months. Donors were instructed to collect feces in small containers. Sample collection was approved by the Ethics Committee of Tianjin Medical University General Hospital.

### Co-cultivation of intestinal microorganisms and engineered yeasts

Hemin (Tuopu Biotechnology, China), Wolin’s vitamin solution (Tuopu Biotechnology, China), and volatile fatty acids (Tuopu Biotechnology, China) were added to the YCFA basal medium to prepare the YCFA medium.

To culture the samples, 1 mL of YCFA was placed in 96-deep well plates (LabSelect, China). The plates were then covered with a silicone gel mat. Fresh stool samples were collected from each patient and immediately transferred to an anaerobic workstation (37°C) containing 5% H_2_, 5% CO_2_, and 90% N_2_. The samples were dissolved in a Falcon tube containing 10 mL of PBS. Prior to homogenization using a vortex mixer, the tube was briefly uncapped to allow gas exchange and oxygen removal. Homogenized samples were filtered through sterile gauze and promptly inoculated into the medium at a final concentration of 2% (w/v). Engineered yeast was added to a 2% inoculum for the intervention. The cultures were incubated at 37°C with constant shaking at 500 rpm using digital shakers for 24 hours.

### Mice

60 male C57BL/6 mice (8–10 weeks old; specific pathogen free, SPF) were purchased from Vital River (Beijing, China). The mice used in this study were housed at Tianjin University following the guidelines set by the Institutional Animal Care and Use Committee. Each cage accommodated two to five mice and maintained a standard cycle of 12 h light and 12 h darkness. The housing conditions were maintained at a temperature of 20–23°C and approximate humidity of 50%. The mice had unrestricted access to food and water. Upon arrival, the mice were acclimatized, and their microbiomes were homogenized over a period of one month. Subsequently, the mice were randomly assigned to different treatment groups to ensure that the mice treated with different probiotic strains were not co-housed to prevent transfer between them.

### Gavage treatment

The yeast was cultivated using YPD medium until OD_600_ = 10, then yeast culture medium was centrifuged at 6500 rpm for 10 minutes until the supernatant and yeast cells were separated, and supernatant was discarded. An appropriate amount of sterile PBS water was added to the yeast cell precipitate, and the precipitate was resuspended to allow the yeast and water to come into full contact. The resuspended liquid was centrifuged at 6500 rpm for 10 minutes, and the supernatant was removed. This step was repeated to remove residual culture medium and butyrate from yeast precipitation. Subsequently, an appropriate amount of PBS water was added to the cleaned cell precipitate. The cell precipitate was resuspended again to achieve a cell concentration of 10^9^ CFU/mL. The prepared yeast resuspension was placed at 4°C for storage. Each group was set up with 10 mice. After the adaptation period ended, each group of mice was given 200 μL of PBS, yeast cell solution by gavage, once a day. After continuous gavage for two weeks, modeling began. Each mouse in the Control group and TNBS group was given 200 μL of PBS by gavage, while each mouse in the administration group was given 200 μL of engineered yeast solution with a concentration of 10^9^ CFU/mL by gavage.

### Trinitrobenzenesulfonic acid-induced mouse colitis model

To induce colitis in C57BL/6J mice using TNBS, male mice were sensitized one week prior to colitis induction. The sensitization involved applying 150 μL of TNBS solution (composed of 64% acetone from Sigma-Aldrich, 16% olive oil from Sigma-Aldrich, and 20% TNBS solution at a concentration of 50 mg/mL (picrylsulfonic acid solution, 5%, Sigma-Aldrich)) to their preshaved backs. After one week, the sensitized mice were fasted for 4 hours and then received 100 µl of the TNBS induction solution (composed of 50% ethanol and 50% TNBS solution at a concentration of 50 mg/mL) rectally. The control group was administered 50% ethanol. The weights of the mice were monitored daily until euthanasia was performed at the peak of the disease after colitis induction.

### Disease activity index

The Disease Activity Index (DAI) was assessed based on the following parameters: stool consistency (scored as 0 for hard, 2 for soft, and 4 for diarrhea), fecal occult blood using Hemoccult Sensa (Beckman Coulter) (scored as 0 for negative, 2 for positive, and 4 for macroscopic), and weight loss (scored as 0 for less than 1%, 1 for 1–5%, 2 for 5–10%, 3 for 10–20%, and 4 for more than 20%).

### Hematoxylin and eosin (H&E) staining

The colonic segments, approximately 2–3 cm in length, were surgically removed, washed with phosphate-buffered saline (PBS), fixed in 4% formaldehyde, embedded in paraffin, and then sectioned into slices 5 μm thick. One set of paraffin sections was stained with hematoxylin and eosin (H&E) staining. The total damage score was determined based on several criteria including goblet cell depletion (scored as 1 for presence and 0 for absence), crypt abscesses (scored as 1 for presence and 0 for absence), destruction of mucosal architecture (scored as 1 for normal, 2 for moderate, and 3 for extensive), muscle thickening (scored as 1 for normal, 2 for moderate, and 3 for extensive), and cellular infiltration (scored as 1 for normal, 2 for moderate, and 3 for transmural).^100^

### TNF-α, IL-6 and IL-1β secretion assays

Enzyme-linked immunosorbent assay kits (ELISA, SBJ-M0030, SenBeiJia Biological Technology) (ELISA, SBJ-M0657, SenBeiJia Biological Technology) (ELISA, SBJ-R0546, SenBeiJia Biological Technology) were used to detect the secretion of TNF-α, IL-6 and IL-1β in cell cultures according to the manufacturer’s instructions. Briefly, supernatant of primary cardiomyocytes with different treatment was collected and centrifuged (500 g) for 5 minutes. Then, the supernatant was sub-jected to ELISA assay.

### RNA extraction and quantitative PCR

A sample weighing 20 mg was taken from the distal colon and immediately flash-frozen. The frozen samples were disrupted using TRIzol reagent (Invitrogen) for RNA extraction. The RNA extraction process followed the guidelines provided by the manufacturer’s miRNeasy kit (Qiagen, Germany). Reverse transcription was performed to convert RNA into cDNA using the PrimeScript™ RT reagent kit (TaKaRa). Then, cDNA was amplified using the ChamQ Universal SYBR qPCR Master Mix (Vazyme, China) to confirm the expression levels of the genes. The calculation method of transcript level was through the -ΔΔCt method.^101^

### Statistical analysis

Statistical analyses were conducted using Prism 9 software. Student’s t-test and one-way analysis of variance (ANOVA) analysis were employed, followed by Tukey’s or Fisher’s least significant difference multiple comparisons. Statistical significance was determined at a threshold of p < 0.05. The following notation was used to denote significance levels: (n.s.) for p > 0.05, * for p < 0.05, ** for p < 0.01, *** for p < 0.001, and **** for p < 0.0001. Results with *p* values above 0.05 were considered not significant (n.s.).

## Supplementary Material

Revised supplemental information clean.docx

## Data Availability

The 16S rRNA gene sequencing raw data generated in this study have been deposited in the NCBI under accession No. PRJNA998236. Other data needed to evaluate the conclusions in the paper are present in the paper and/or the Supplementary Materials.

## Abbreviations

IBD: Inflammatory bowel disease
SCFA: Short-chain fatty acid
GPD: Glycerol-3-phosphate dehydrogenase
DSS: Dextran sulfate sodium
UC: Ulcerative colitis
ACE: Abundance Coverage-based Estimator
DAI: Disease activity index
TNBS: Trinitro-benzene-sulfonic acid
BA: Butyric acid
TNF: Tumor necrosis factor
IL: Interleukin
ATP: Adenosine triphosphate
NADH: Nicotinamide adenine dinucleotide
HC: Healthy Control
CON: Control
eATP: Extracellular Adenosine triphosphate
