# Supplementary material for: A tailored series of engineered yeasts for the cell-dependent treatment of inflammatory bowel disease by rational butyrate supplementation
Source: Gut Microbes. 2024 Feb 21;16(1):2316575. doi: 10.1080/19490976.2024.2316575 (PMC10883098; doi:10.1080/19490976.2024.2316575)
Supplement: Revised supplemental information clean.docx [file KGMI_A_2316575_SM1353.docx]

**Supplementary materials**

**Materials and Reagents**

**Plasmids construction**

All the plasmids and primers used in this study are shown in Table. S1. The plasmids were constructed according to the manufacturer's instruction of the In-fusion HD cloning Kit purchased from Takara (catalogue # 638911). *Clostridium beijerinckii Hbd* and *Crt*, *Treponema denticola* *Ter*, *Faecalibacterium prausnitzii HADH*, *ECHA*, *CCRA and BCoAT*, and *E. coli atoDA* were codons optimised for *S. cerevisiae* and synthesised by Genewiz.

The plasmids (pESC-His3-Hbd-Crt, pESC-Ura3-Ter, pESC-Leu2-atoD, pESC-Leu2-BCoAT, pESC-His3-HADH-ECHA and pESC-Ura3-CCRA) expressing Hbd and Crt, Ter, atoDA, BCoAT, HADH and ECHA, and CCRA were constructed using the Hbd-F/Hbd-R and Crt-F/Crt-R, Ter-F/Ter-R, atoDA-F/atoDA-R, BCoAT-F/BCoAT-R, HADH-F/HADH-R and ECHA-F/ECHA-R, and CCRA-F/CCRA-R primers to amplify *Hbd* and *Crt*, *Ter*, *atoDA*, *BCoAT*, *HADH* and *ECHA*, and *CCRA* fragments from synthetic genes by polymerase chain reaction (PCR), respectively. Then, *Hbd* and *Crt*, *Ter*, *atoDA*, *BCoAT*, *HADH* and *ECHA*, and *CCRA* fragments were cloned into pESC-His3, pESC-Ura3 and pESC-Leu2 to connect the *GAL1* and *GAL10* promoters.

**Strains construction**

JWY1 was constructed with the transformation of pESC-His3-Hbd-Crt, pESC-Ura3-Ter and pESC-Leu2-atoD into BY4741 by the lithium acetate method. JWY2 was constructed with the transformation of pESC-His3-Hbd-Crt, pESC-Ura3-Ter and pESC-Leu2-BCoAT into BY4741. JWY3 was constructed with the transformation of pESC-His3-HADH-ECHA, pESC-Ura3-CCRA and pESC-Leu2-BCoAT into BY4741. JWY4 was constructed with the transformation of pESC-His3-HADH-ECHA, pESC-Ura3-Ter and pESC-Leu2-BCoAT into BY4741. JWY5 was constructed with the transformation of pESC-His3-Hbd-Crt, pESC-Ura3-CCRA and pESC-Leu2-BCoAT into BY4741.

To integrate *Hbd* and *Crt* genes into the genome, *Hbd* and *Crt* fragments were amplified from the pESC-His3-Hbd-Crt plasmid by Hbd-F/Hbd-R and Crt-F/Crt-R primers. *PGK1* and *TPI1* promoter fragments and *PGK1* and *ADH1* terminator fragments were amplified from *S. cerevisiae* BY4741 genome DNA by PCR using the PGK1p-F/PGK1p-R, TPI1p-F/TPI1p-R, PGK1t-F/PGK1t-R, ADH1t-F/ADH1t-R primers. Homologous arms were amplified from the genome of BY4741. Overlap extension PCR was used to get the Hbd-Crt expression cassette by assembling *Hbd* and *Crt* fragments, *PGK1* and *TPI1* promoter fragments, *PGK1* and *ADH1* terminator fragments, and homologous arms. Then Hbd-Crt expression cassette, guide plasmid pRS42H-gRNA (Table 1), and YCplac33-Cas9 (Table 1) were simultaneously transformed into strain BY4741. By using SD-URA screening plates with hygromycin B at the final concentration of 450 μg/mL, positive clones were identified. To integrate *Ter* and *BCoAT* genes into the genome, *Ter* and *BCoAT* fragments were amplified from the pESC-Ura3-Ter and pESC-Leu2-BCoAT plasmids by Ter-F/Ter-R and BCoAT-F/BCoAT-R primers. *PGK1* and *TPI1* promoter fragments, *PGK1* and *ADH1* terminator fragments were amplified from *S. cerevisiae* BY4741 genome DNA by PCR using the PGK1p-F/PGK1p-R, TPI1p-F/TPI1p-R, PGK1t-F/PGK1t-R, ADH1t-F/ADH1t-R primers. Homologous arms were amplified from the genome of BY4741. Ter-BCoAT expression cassette was constructed by assembling *Ter* and *BCoAT* fragments, *PGK1* and *TPI1* promoter fragments, *PGK1* and *ADH1* terminator fragments, and homologous arms. Then, the Ter-BCoAT expression cassette, guide plasmid pRS42H-gRNA, and YCplac33-Cas9 were transformed into the recombinant strain, and transformants were selected on the SD-URA screening plates with hygromycin B.

To delete the *MLS1*, *CIT2*, *ACH1*, *ADH1*, *ADH4*, *GPD1*, *GPD2*, *FAA1*, *FAT1* and *FAA4* genes, the upstream and downstream homologous arms (500bp) of *MLS1*, *CIT2*, *ACH1*, *ADH1*, *ADH4*, *GPD1*, *GPD2*, *FAA1*, *FAT1* and *FAA4* were both amplified from the BY4741 genome by using primers ΔMLS1-H1-F/ΔMLS1-H1-R and ΔMLS1-H2-F/ΔMLS1-H2-R, ΔCIT2-H1-F/ΔCIT2-H1-R and ΔCIT2-H2-F/ΔCIT2-H2-R, ΔACH1-H1-F/ΔACH1-H1-R and ΔACH1-H2-F/ΔACH1-H2-R, ΔADH4-H1-F/ΔADH4-H1-R and ΔADH4-H2-F/ ΔADH4-H2-R, ΔADH1-H1-F/ΔADH1-H1-R and ΔADH1-H2-F/ΔADH1-H2-R, ΔGPD1-H1-F/ΔGPD1-H1-R and ΔGPD1-H2-F/ΔGPD1-H2-R, ΔGPD2-H1-F/ΔGPD2-H1-R and ΔGPD2-H2-F/ΔGPD2-H2-R, ΔFAA1-H1-F/ΔFAA1-H1-R and ΔFAA1-H2-F/ΔFAA1-H2-R, ΔFAA4-H1-F/ΔFAA4-H1-R and ΔFAA4-H2-F/ΔFAA4-H2-R, ΔFAT1-H1-F/ΔFAT1-H1-R and ΔFAT1-H2-F/ΔFAT1-H2-R. The knock-out boxes were formed by ligating the upstream and downstream homologous arms of *MLS1*, *CIT2*, *ACH1*, *ADH1*, *ADH4*, *GPD1*, *GPD2*, *FAA1*, *FAT1* and *FAA4* via overlap extension PCR. Then, knock-out boxes, pRS42H-gRNA and YCplac33-Cas9 were transformed into yeast to generate recombinant strains.

To overexpress *ERG10* and *MCT1* genes, *ERG1*0 and *MCT1* genes fragments were amplified from the genome of BY4741 by PCR using ERG10-F/ERG10-R and MCT1-F/MCT1-R primers. *TEF1* promoter and *TEF1* terminator fragments, *TDH3* promoter and *TDH3* terminator fragments were amplified from the genome of BY4741. After homologous arms were amplified from the genome of BY4741, ERG10 or ERG10-MCT1 cassette was constructed by using overlap extension PCR to assemble homologous arms, *ERG10* and *MCT1* genes fragments, *TEF1* promoter and *TEF1* terminator fragments, *TDH3* promoter and *THD3* terminator fragments. Afterwards, the cassette was transformed into recombinant strains with pRS42H-gRNA and YCplac33-Cas9. To overexpress *ACS1*, *ACS2* and *ALD6* genes, *ACS1*, *ACS2* and *ALD6* genes fragments were amplified from the BY4741 genome by PCR using ACS1-F/ACS1-R, ACS2-F/ACS2-R and ALD6-F/ ALD6-R. *TEF1* promoter and *TEF1* terminator fragments, *TDH3* promoter and *TDH3* terminator fragments were amplified from the BY4741 genome. ACS1 or ACS1-ACS2 or ACS1-ACS2-ALD6 cassette was constructed by using overlap extension PCR to assemble homologous arms, *ERG10* and *MCT1* genes fragments, *TEF1* promoter and *TEF1* terminator fragments, *TDH3* promoter and *THD3* terminator fragments. Then the cassette was transformed into yeast to generate a recombinant strain with pRS42H-gRNA and YCplac33-Cas9.


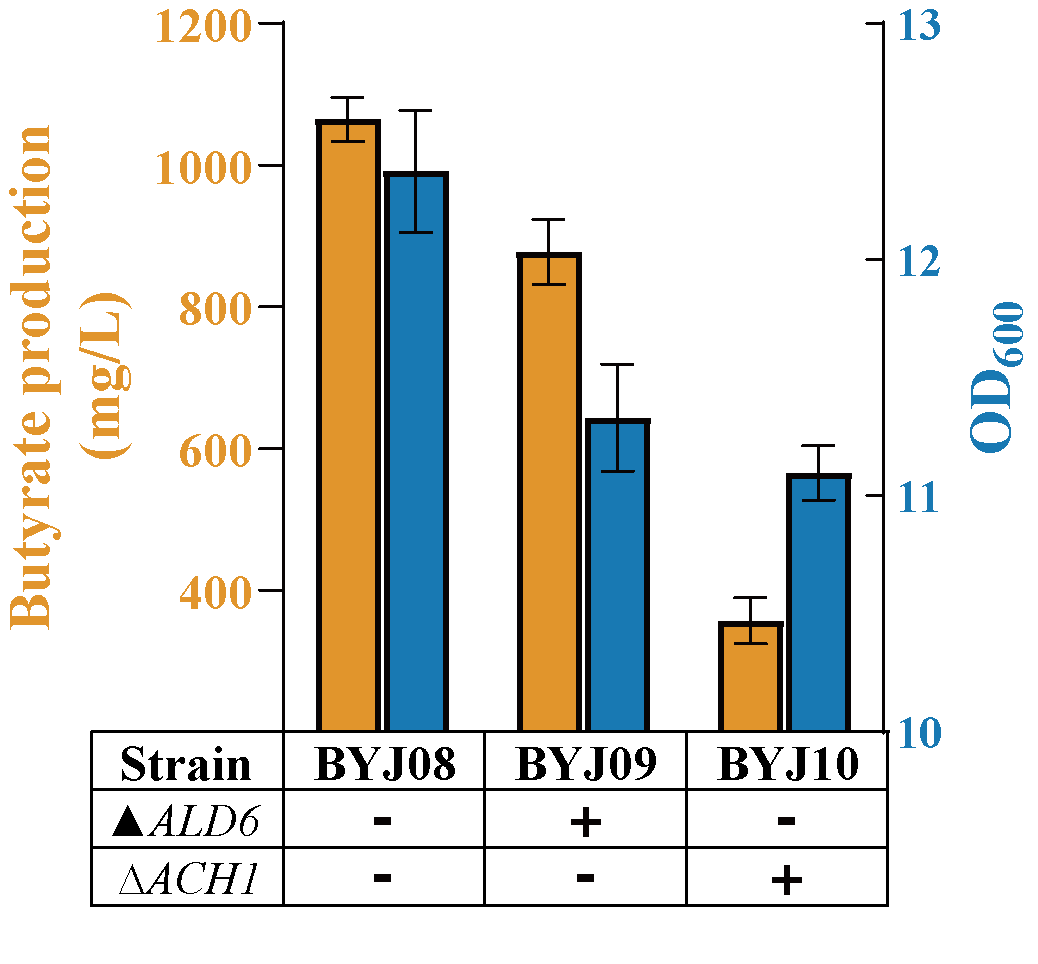


**Fig. S1. Butyrate production and vitality of engineered yeasts.** BYJ09, with *ALD6* overexpressed; BYJ10, with *ACH1* deleted.


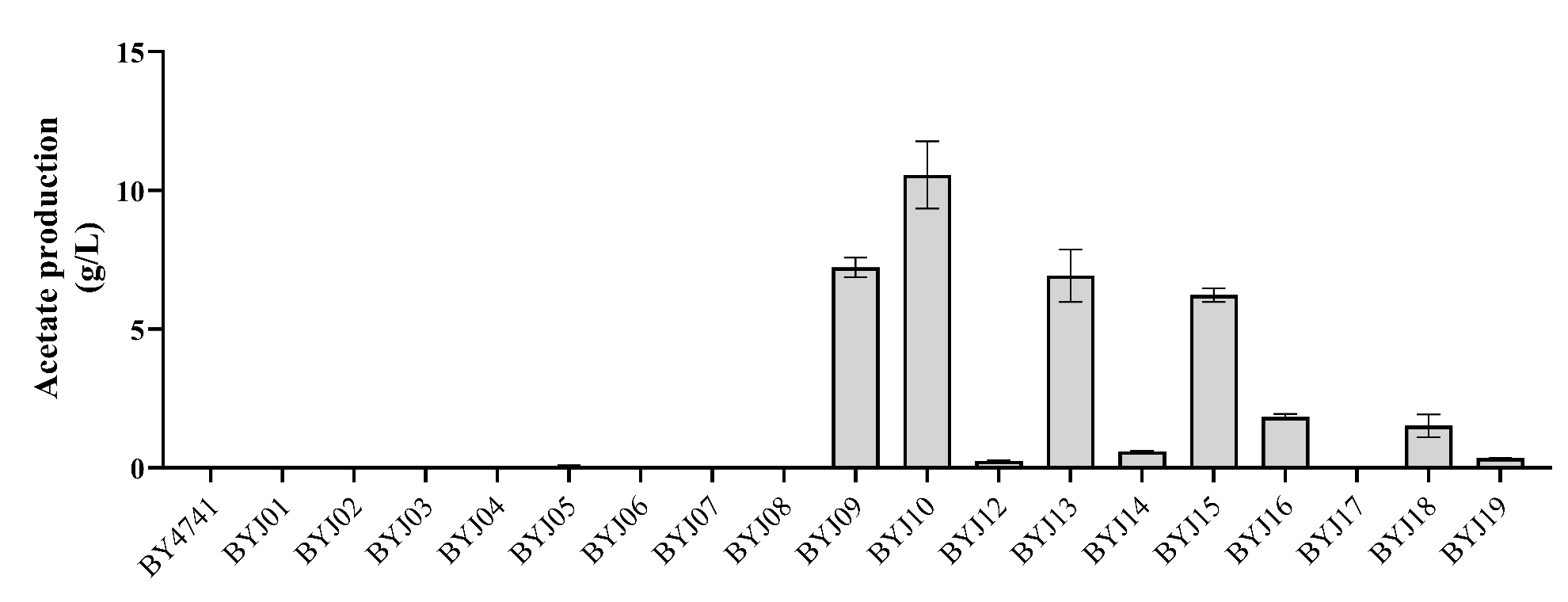
**Fig. S2. Acetate production of different engineered yeasts.**


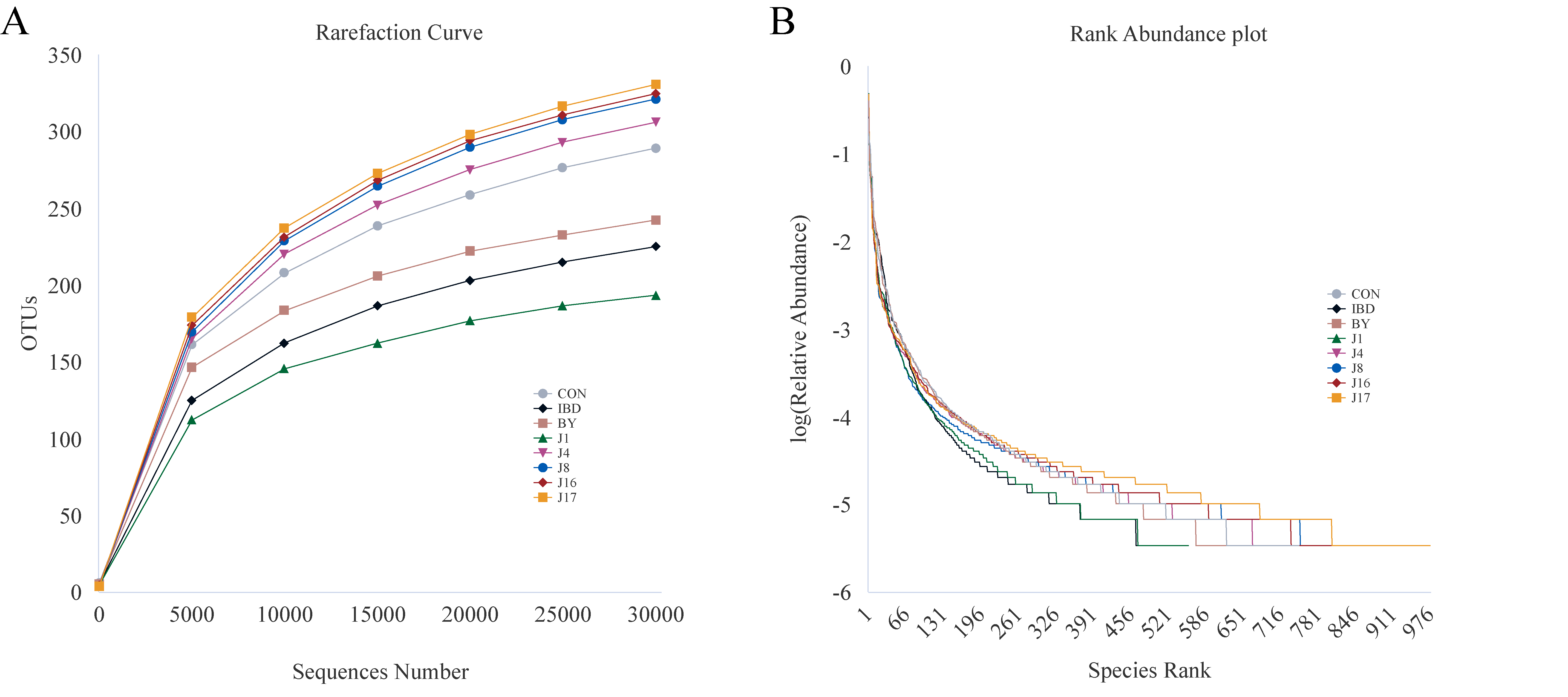


**Fig. S3. Diversity curves of different groups of gut microbiota.** (**A**) Rarefaction Curve of different groups. (**B**) Rank Abundance of different groups.


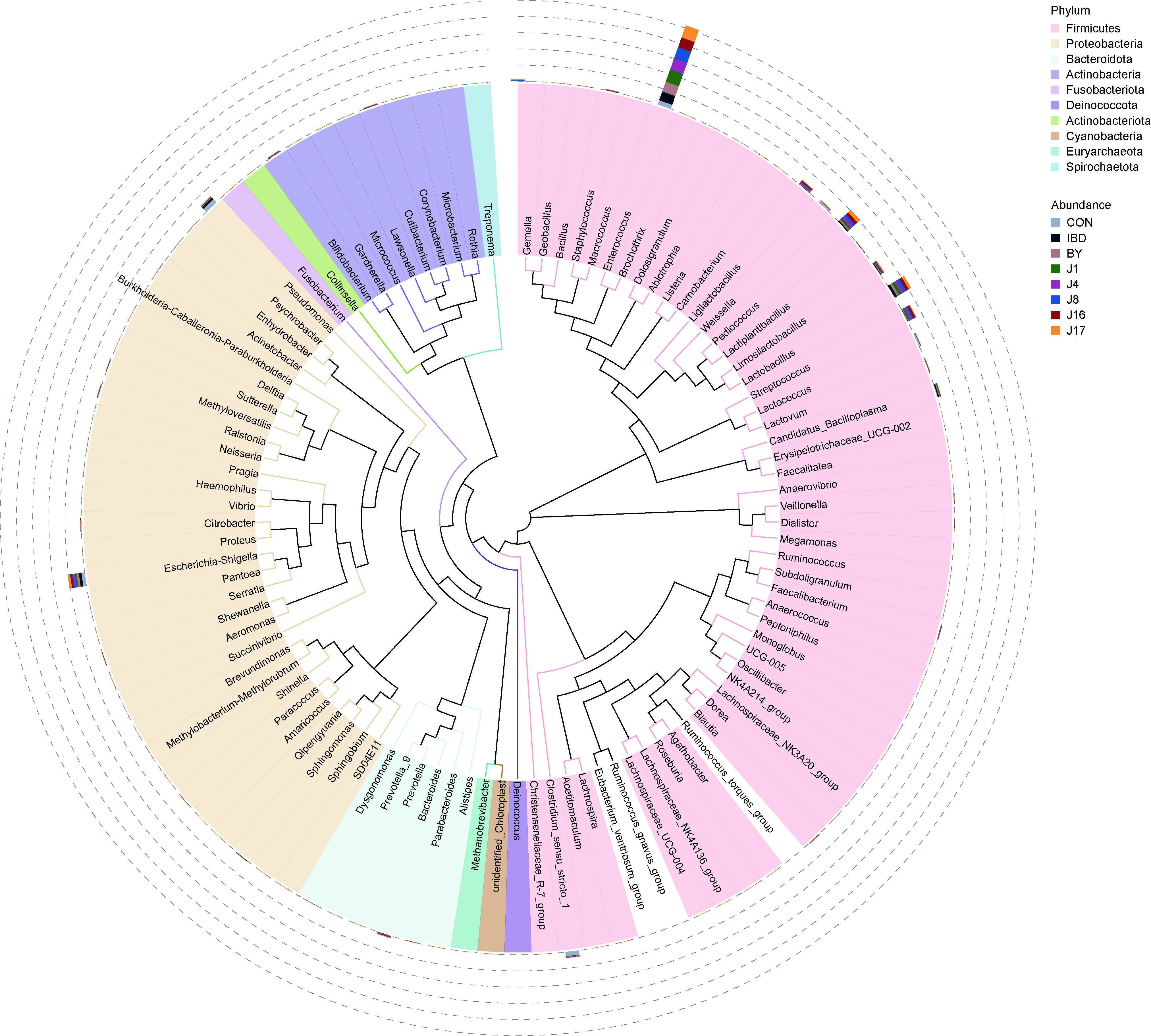


**Fig. S4. Genus-level phylogenetic tree of gut microbiota.**


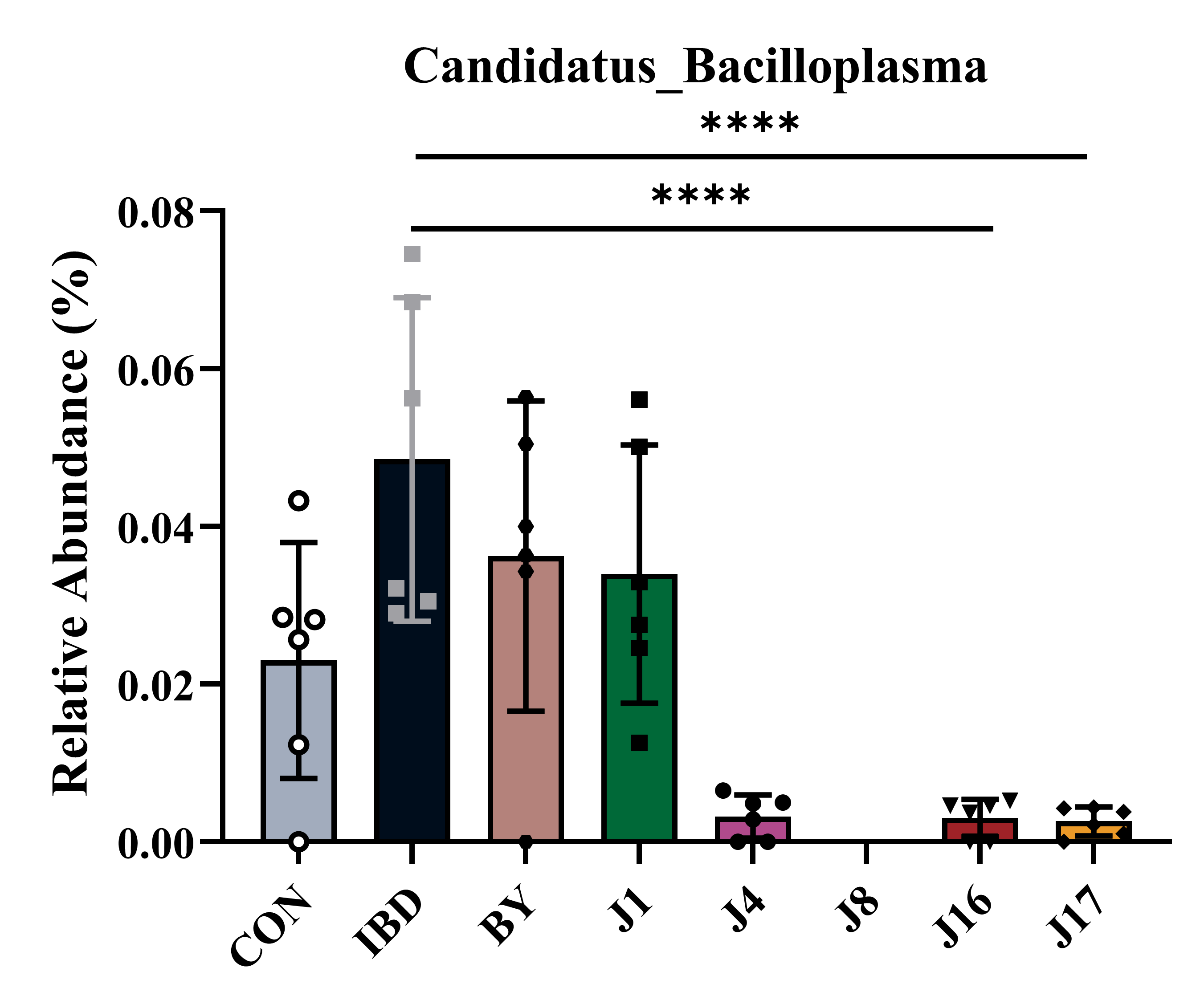


**Fig. S5. Relative abundance of Candidatus_Bacilloplasma at the species level in the gut microbiota.**


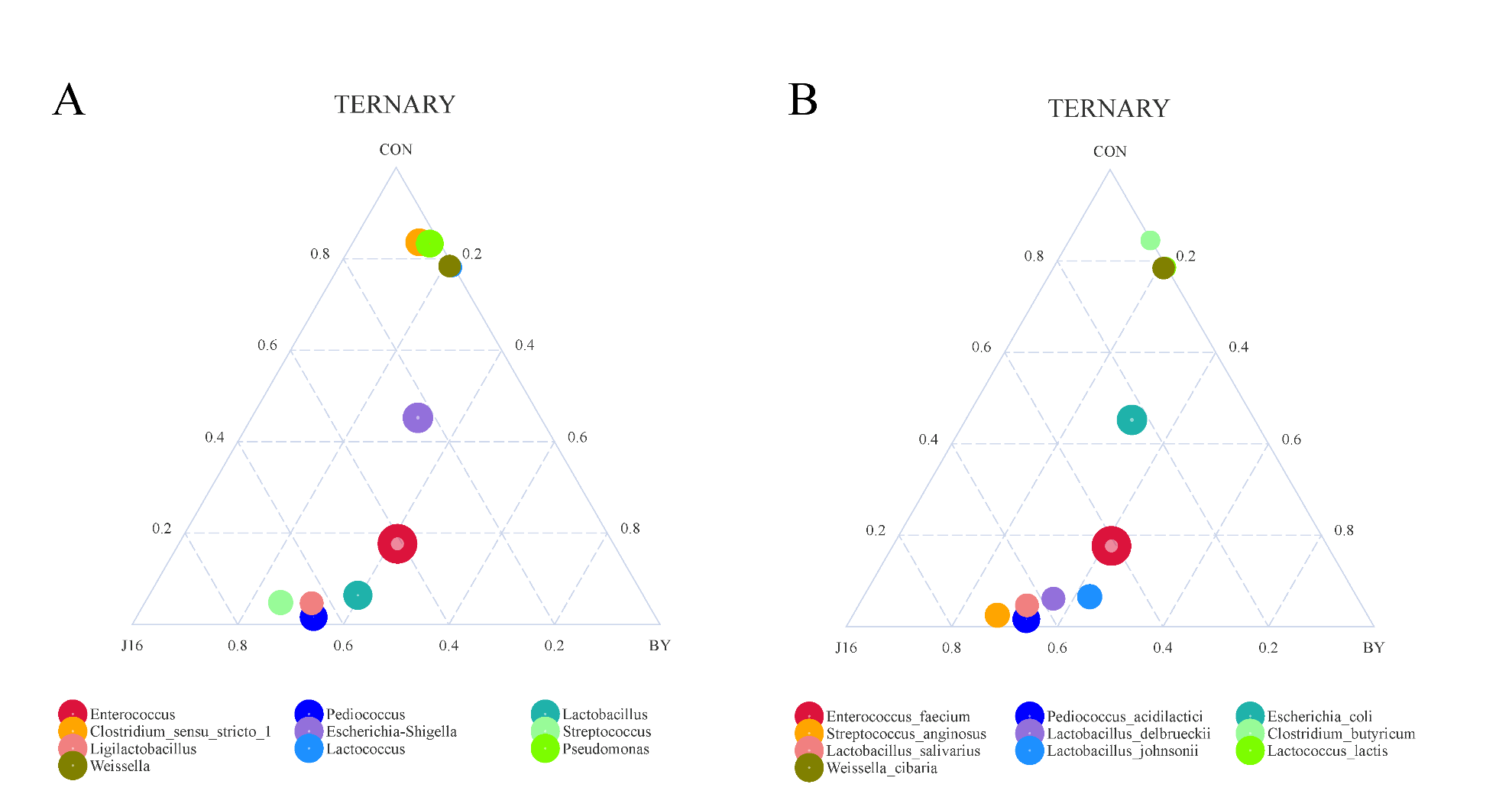


**Fig. S6. The genus** (**A**) **and species** (**B**) **level dominant microorganisms of the J16 and BY treatment groups and the CON group are shown in the ternary diagram.**

**Table S1. Strains and plasmids used in this study.**

| Strain/plasmid | Relevant features/genotype | Resource |
| --- | --- | --- |
| Plasmids |  |  |
| YCplac33 | *Ampr, URA3* | Addgene |
| p415-Cas9 | *Ampr, LEU2, Cas9* | Addgene |
| YCplac33-Cas9 | *Ampr*, *URA3*, *Cas9* | This work |
| pRS42H-dCas9 | *Ampr, hph* NT1 | Addgene |
| p426-Cas9-gRNA | *Ampr*, *URA3*, crRNA | Addgene |
| pRS42H-gRNA | *Ampr*, *hph* NT1, crRNA | This work |
| pESC-His3-HADH-ECHA | pESC-*His3*-P*_GAL1_*-*HADH*/P*_GAL10_*-*ECHA* | This work |
| pESC-His3-Hbd-Crt | pESC-*His3*-P*_GAL1_*-*Hbd*/P*_GAL10_*-*Crt* | This work |
| pESC-Ura3-CCRA | pESC-*Ura3*-P*_GAL1_*-*CCRA* | This work |
| pESC-Ura3-Ter | pESC-*Ura3*-P*_GAL1_*-*Ter* | This work |
| pESC-Leu2-atoD | pESC-*Leu2*-P*_GAL1_*-*atoD* | This work |
| pESC-Leu2-BCoAT | pESC-*Leu2*-P*_GAL1_*-*BCoAT* | This work |
| Strains with plasmid |  |  |
| JWY1 | BY4741: pESC-*His3*-*Hbd*-*Crt*+pESC-*Ura3*-*Ter*+pESC-*Leu2*-*atoD* | This work |
| JWY2 | BY4741: pESC-*His3*-*Hbd*-*Crt*+pESC-*Ura3*-*Ter*+pESC-*Leu2*-*BCoAT* | This work |
| JWY3 | BY4741: pESC-*His3*-*HADH*-*ECHA*+pESC-*Ura3*-*CCRA*+pESC-*Leu2*-*BCoAT* | This work |
| JWY4 | BY4741: pESC-*His3*-*HADH*-*ECHA*+pESC-*Ura3*-*Ter*+pESC-*Leu2*-*BCoAT* | This work |
| JWY5 | BY4741: pESC-*His3*-*Hbd*-*Crt*+pESC-*Ura3*-*CCRA*+pESC-*Leu2*-*BCoAT* | This work |
| Strains without plasmid |  |  |
| *S. cerevisiae* BY4741 | *ΔMAT*; *his3Δ*； l*eu2Δ*; *met15Δ*; *ura3Δ* | ATCC |
| BYJ01 | *ΔMAT*; *his3Δ*; *leu2Δ*; *met15Δ*; *ura3Δ*; *hbd*::P*_PGK1_*-*Hbd*; *Crt*::P*_TPI1_*-*Crt*; *ter*::P*_PGK1_*-*TER*; *bcoat*::P*_TPI1_*-*BCoAT* | This work |
| BYJ02 | BYJ01; *erg10*:: P*_TEF1_*-*ERG10* | This work |
| BYJ03 | BYJ01; *mct1*:: P*_TDH3_*-*MCT1* | This work |
| BYJ04 | BYJ01; *erg10*:: P*_TEF1_*-*ERG10* ; *mct1*::P*_TDH3_*-*MCT1* | This work |
| BYJ05 | BYJ04; *MLS1Δ* | This work |
| BYJ06 | BYJ04; *MLS1Δ*; *CIT2Δ* | This work |
| BYJ07 | BYJ04; *MLS1Δ*; *CIT2Δ*; *acs1*:: P*_TEF1_*-*ACS1* | This work |
| BYJ08 | BYJ04; *MLS1Δ*; *CIT2Δ*; *acs1*::P*_TEF1_*-*ACS1*; *acs2*::P*_TEF1_*-*ACS2* | This work |
| BYJ09 | BYJ04; *MLS1Δ*; *CIT2Δ*; *acs1*:: P*_TEF1_*-*ACS1*; *acs2*::P*_TEF1_*-*ACS2*; *ald6*::P*_TDH3_*-*ALD6* | This work |
| BYJ10 | BYJ04; *MLS1Δ*; *CIT2Δ*; *acs1*:: P*_TEF1_*-*ACS1*; *acs2*::P*_TEF1_*-*ACS2*; *ACH1Δ* | This work |
| BYJ12 | BYJ04; *MLS1Δ*; *CIT2Δ*; *acs1*:: P*_TEF1_*-*ACS1*; *acs2*::P*_TEF1_*-*ACS2*; *ADH4Δ* | This work |
| BYJ13 | BYJ04; *MLS1Δ*; *CIT2Δ*; *acs1*:: P*_TEF1_*-*ACS1*; *acs2*::P*_TEF1_*-*ACS2*; *ADH1Δ*; *ADH4Δ* | This work |
| BYJ14 | BYJ04; *MLS1Δ*; *CIT2Δ*; *acs1*:: P*_TEF1_*-*ACS1*; *acs2*::P*_TEF1_*-*ACS2*; *GPD1Δ* | This work |
| BYJ15 | BYJ04; *MLS1Δ*; *CIT2Δ*; *acs1*:: P*_TEF1_*-*ACS1*; *acs2*::P*_TEF1_*-*ACS2*; *GPD1Δ*; *ADH1Δ*; *ADH4Δ* | This work |
| BYJ16 | BYJ04; *MLS1Δ*; *CIT2Δ*; *acs1*::P*_TEF1_*-*ACS1*; *acs2*::P*_TEF1_*-*ACS2*; *ADH1Δ*; *ADH4Δ*; *GPD1Δ*; *GPD2Δ* | This work |
| BYJ17 | BYJ04; *MLS1Δ*; *CIT2Δ*; *acs1*::P*_TEF1_*-*ACS1*; *acs2*::P*_TEF1_*-*ACS2*; *ADH1Δ*; *ADH4Δ*; *GPD1Δ*; *GPD2Δ*; *FAA1Δ* | This work |
| BYJ18 | BYJ04; *MLS1Δ*; *CIT2Δ*; *acs1*::P*_TEF1_*-*ACS1*; *acs2*::P*_TEF1_*-*ACS2*; *ADH1Δ*; *ADH4Δ*; *GPD1Δ*; *GPD2Δ*; *FAA4Δ* | This work |
| BYJ19 | BYJ04; *MLS1Δ*; *CIT2Δ*; *acs1*::P*_TEF1_*-*ACS1*; *acs2*::P*_TEF1_*-*ACS2*; *ADH1Δ*; *ADH4Δ*; *GPD1Δ*; *GPD2Δ*; *FAT1Δ* | This work |
| SCB-1 | BYJ01; *MLS1Δ* | This work |
| SCB-2 | BYJ01; *MLS1Δ*; *CIT2Δ* | This work |
| SCB-3 | BYJ01; *MLS1Δ*; *CIT2Δ*; *acs1*::P*_TEF1_*-*ACS1* | This work |
| SCB-4 | BYJ01; *MLS1Δ*; *CIT2Δ*; *acs1*::P*_TEF1_*-*ACS1*; *acs2*::P*_TEF1_*-*ACS2* | This work |
| SCB-5 | BYJ01; *GPD1Δ* | This work |
| SCB-6 | BYJ01; *ADH4Δ* | This work |
| SCB-7 | BYJ01; *FAA1Δ* | This work |

**Table S2. Primers used for gene editing and gene expression levels in yeast.**

| **Primer** | **Sequence (5' to 3')** |
| --- | --- |
| Hbd-F | ATGAAGAAGGTTTGTGTTAT |
| Hbd-R | TTACTTAGAGTAATCGTAGA |
| Crt-F | ATGGAATTGAATAACGTTAT |
| Crt-R | TTATCTATTCTTAAAACCTT |
| Ter-F | ATGATTGTTAAGCCAATGGT |
| Ter-R | TTAAATTCTATCGAATCTTT |
| BCoAT-F | ATGGATTTTACTGAATTGTA |
| BCoAT-R | TTAATGATGATGATGATGAT |
| ERG10-F | ATGTCTCAGAACGTTTACAT |
| ERG10-R | TCATATCTTTTCAATGACAA |
| MCT1-F | ATGAAGCTACTAACCTTCCC |
| MCT1-R | TCAATCTTTGTTCTCCTCTG |
| ACS1-F | ATGTCGCCCTCTGCCGTACA |
| ACS1-R | TTACAACTTGACCGAATCAA |
| ACS2-F | ATGACAATCAAGGAACATAA |
| ACS2-R | TTATTTCTTTTTTTGAGAGA |
| ALD6-F | ATGACTAAGCTACACTTTGA |
| ALD6-R | TTACAACTTAATTCTGACAG |
| ΔMLS1-H1-F | GCCTCCATTGAGAAATATGC |
| ΔMLS1-H1-R | AAGGTGCTTTTCCACCATGTCGGGGTCTCACGATAATAGT |
| ΔMLS1-H2-R | ACTATTATCGTGAGACCCCGACATGGTGGAAAAGCACCTT |
| ΔMLS1-H2-R | CACTAGAATGTTGTCTTAAT |
| ΔCIT2-H1-F | TTCGTTTCAGAGGTCGTACG |
| ΔCIT2-H1-R | GCAGTTACAGCAATAGAGAATTGGGTGTAAGTCCTTTGGT |
| ΔCIT2-H2-F | ACCAAAGGACTTACACCCAATTCTCTATTGCTGTAACTGC |
| ΔCIT2-H2-R | ACCACCTTCGTGATCCGAAT |
| ΔACH1-H1-F | GAGGAAAACCGTTGGGCTGA |
| ΔACH1-H1-R | CGACAGAGGGACCTGGGACGGTCCTCTTTAATGGCCGTTG |
| ΔACH1-H2-F | CAACGGCCATTAAAGAGGACCGTCCCAGGTCCCTCTGTCG |
| ΔACH1-H2-R | TCGACCAAATGACCTGCAAT |
| ΔADH4-H1-F | GCGCACTACTTGAAAGGTTA |
| ΔADH4-H1-R | GTTTTGTTCCTTCAAAACCTGCTGTGACATTGGCAATATT |
| ΔADH4-H2-F | AATATTGCCAATGTCACAGCAGGTTTTGAAGGAACAAAAC |
| ΔADH4-H2-R | GGCATCATGCATGGCGTGTT |
| ΔADH1-H1-F | CGGAGGGGAGATATACAATA |
| ΔADH1-H1-R | GACAACGACACCGGCACCTTCCGACTAATGGTAGCTTAAC |
| ΔADH1-H2-F | GTTAAGCTACCATTAGTCGGAAGGTGCCGGTGTCGTTGTC |
| ΔADH1-H2-R | CGTATCTACCAACGATTTGA |
| ΔGPD1-H1-F | GTATATTGTACACCCCCCCC |
| ΔGPD1-H1-R | ACCCCAGCCTAGACCTTCGACAACCTAAGGCAACAACGTT |
| ΔGPD1-H2-F | AACGTTGTTGCCTTAGGTTGTCGAAGGTCTAGGCTGGGGT |
| ΔGPD1-H2-R | GCTCTTTGGAACTTGTCCAT |
| ΔGPD2-H1-F | GCAGCTCTTCTCTACCCTGT |
| ΔGPD2-H1-R | CACGAGATGGCCCTTACATGCGTGGCCTTGCAATTGTTTG |
| ΔGPD2-H2-F | CAAACAATTGCAAGGCCACGCATGTAAGGGCCATCTCGTG |
| ΔGPD2-H2-R | GGGGGAAAAAGAGGCAACAG |
| ΔFAA1-H1-F | GCAGCCCTGTCATATTCTTC |
| ΔFAA1-H1-R | TCATTAGGCTTTAATCCTATACCCACGACCAATTTCATGC |
| ΔFAA1-H2-F | GCATGAAATTGGTCGTGGGTATAGGATTAAAGCCTAATGA |
| ΔFAA1-H2-R | GGTGAGGAAGGGCAAATTAT |
| ΔFAA4-H1-F | GCCGTTTCCCAACAAAAAAC |
| ΔFAA4-H1-R | AACACCAATCTTTATCAGCCATGTCGTGCATTACGCAGAT |
| ΔFAA4-H2-F | ATCTGCGTAATGCACGACATGGCTGATAAAGATTGGTGTT |
| ΔFAA4-H2-R | GAGACCAACCGGGCAATTCG |
| ΔFAT1-H1-F | GGGATAGGAAAAGCTTGAAA |
| ΔFAT1-H1-R | TCGAGTTGAAATTCTCCCTTTGGGACGGGTGTAACTAATC |
| ΔFAT1-H2-F | GATTAGTTACACCCGTCCCAAAGGGAGAATTTCAACTCGA |
| ΔFAT1-H2-R | TAAGCAACCTTCACCTTATG |
| PGK1-F | GGGCCAGAAAAAGGAAGTGT |
| PGK1-R | TGTTTTATATTTGTTGTAAA |
| TPI1-F | TAACGGGAGCGTAATGGTGA |
| TPI1-R | TTTTAGTTTATGTATGTGTT |
| TEF1-F | GAAGGTTCACGAAATCTTTA |
| TEF1-R | TTTGTAATTAAAACTTAGAT |
| TDH3-F | ATACTAGCGTTGAATGTTAG |
| TDH3-R | TTTGTTTGTTTATGTGTGTT |
| hbd-a | TGGCTGCTGATTGTGATTTGG |
| hbd-s | AGCGGAAGCAACTTCGGTAA |
| crt-a | TCGTTGCTGGTGCTGACATT |
| crt-s | ACCACCTAAAGCGAAACCGT |
| ter-a | CCGGTGCTAAGGCTCCAAAA |
| ter-s | TGGTTTCAGAACCGGCCTTT |
| bcoat-a | TCATTTGGGTGCCGTTTGC |
| bcoat-s | CCTTCAACAACACCAGCAACTT |
| acs1-a | TGTTGACCATGCGCTACACT |
| acs1-s | AGACCAAAGTGGCACAACCA |
| acs2-a | ACTTGCATTGGGATGCTCCA |
| acs2-s | CTGGCTTGTCGGGATTAGCA |
| ach1-a | TATCGTCCCAGGTCCCTCTG |
| ach1-s | TCCTGAAAGGTGGGTTCACG |
| act1-a | CAAACCGCTGCTCAATCTTC |
| act1-s | AGTTTGGTCAATACCGGCAG |
|  |  |

| **Primer** | **Sequence (5' to 3')** |
| --- | --- |
| GAPDH-F | GGAGAAACCTGCCAAGTATG |
| GAPDH-R | TGGGAGTTGCTGTTGAAGTC |
| TNF-α-F | CCTGTAGCCCACGTCGTAG |
| TNF-α-R | GGGAGTAGACAAGGTACAACCC |
| IL-6-F | CTGCAAGAGACTTCCATCCAG |
| IL-6-R | AGTGGTATAGACAGGTCTGTTGG |
| IL-1β-F | GAAATGCCACCTTTTGACAGTG |
| IL-1β-R | TGGATGCTCTCATCAGGACAG |
| IL-10-F | TGGACAACATACTGCTAACCG |
| IL-10-R | GGATCATTTCCGATAAGGCT |
| ZO-1-F | GCTTTAGCGAACAGAAGGAGC |
| ZO-1-R | TTCATTTTTCCGAGACTTCACCA |
| MUC-2-F | AGGGCTCGGAACTCCAGAAA |
| MUC-2-R | CCAGGGAATCGGTAGACATCG |

**Table S3. Primers used for detection of relative gene expression level in mice.**

**Table S4. Baseline characteristics of inflammatory bowel disorders (IBD) patients and healthy controls (HC) volunteers.**

|  | IBD | HC |
| --- | --- | --- |
| Subjects, n | 6 | 6 |
| Gender, female/male | 3/3 | 3/3 |
| Median age, years(range) | 41.5（31-59） | 36.5（30-50） |
| Disease activity, n |  |  |
| Active | 6 | - |
| Inactive | 0 | - |
| Mayo score |  |  |
| Mild | 2 | - |
| Moderate | 4 | - |
| Severe | 0 | - |
| Inflammation site |  |  |
| Ileitis | 0 | - |
| Colitis | 6 | - |
| Colitis and ileitis | 0 | - |
| Proctitis | 0 | - |
| Medications, n |  |  |
| Mesalazine | 0 | - |
| Corticosteroids | 0 | - |
| Thiopurines | 0 | - |
| Anti-TNF-α | 0 | - |
